# An Overview of Advanced Materials and Manufacturing Strategies for 3D-Printed Bioengineered Vascular Stents: Toward Next-Generation Drug Delivery Applications

**DOI:** 10.3390/pharmaceutics18060755

**Published:** 2026-06-21

**Authors:** Faisal Khaled Aldawood

**Affiliations:** Department of Industrial Engineering, College of Engineering, University of Bisha, P.O. Box 551, Bisha 61922, Saudi Arabia; faldawood@ub.edu.sa

**Keywords:** 3D printing, additive manufacturing, biomaterials, biomedical applications, drug delivery systems, drug-eluting devices, medical devices, pharmaceutical manufacturing

## Abstract

Additive manufacturing has emerged as a transformative technology for fabricating complex drug-eluting medical devices, offering unprecedented design freedom and functional integration capabilities. This comprehensive review systematically analyzes 3D printing technologies applied to pharmaceutical device manufacturing, focusing on drug-eluting vascular stents as a representative application. This review covers six primary additive manufacturing techniques, ranging from high-resolution vat photopolymerization (25 μm resolution) to direct energy deposition, with a focus on their capabilities for produce pharmaceutical devices with controlled drug release properties. Novel 4D/5D/6D printing technologies introduce stimuli-responsive behaviors enabling programmable drug release profiles and adaptive device functionality. Manufacturing process optimization reveals superior design flexibility compared to conventional methods, with 85–95% reduction in design iteration time and elimination of tooling costs for complex geometries. The material landscape encompasses traditional metals (316L stainless steel, cobalt–chromium), biodegradable polymers (polylactic acid, PLA; polycaprolactone, PCL; poly(lactic-co-glycolic acid), PLGA), shape-memory materials (i.e., polymers and alloys capable of recovering a pre-programmed shape upon exposure to a specific stimulus such as body temperature, moisture, or light), and advanced nanocomposites, each offering distinct drug-loading capacities (100–500 μg/cm^2^) and release kinetics. Critical challenges include standardization requirements (International Organization for Standardization (ISO) 5840 and American Society for Testing and Materials (ASTM) F2606), pharmaceutical-grade manufacturing protocols, and regulatory pathways for novel drug-device combinations. This review identifies key research priorities including development of biocompatible printing materials, accelerated drug release testing protocols, and scalable manufacturing processes suitable for medical device production. This analysis demonstrates that 3D printing enables integration of multiple pharmaceutical functions within single devices, controlled spatiotemporal drug delivery, and elimination of secondary manufacturing steps for drug coating processes, advancing the development of next-generation therapeutic medical devices.

## 1. Introduction

### 1.1. Background and Historical Development of Vascular Stents

Among the most significant innovations in interventional cardiology over the past four decades is the evolution of vascular stent technology [1,2,3,4]. Mechanical scaffolding to maintain vessel patency emerged as a compelling therapeutic strategy [5]. Coronary angiography was developed in the late 1950s, and percutaneous transluminal balloon angioplasty (BA) was introduced in the mid-1960s, laying the foundation for stent technology [6,7,8]. Balloon angioplasty was initially applied to the revascularization of femoral, popliteal, and renal arteries, and was subsequently extended to coronary arteries by the late 1970s [9]. However, balloon angioplasty carried significant limitations: it could produce uncontrollable plaque disruption with subsequent vascular recoil, periprocedural coronary occlusion, and myocardial infarction [10]. Moreover, a 20–40% rate of restenosis was observed within 6–12 months of successful revascularization [11]. Various atherectomy techniques were developed during the late 1980s and early 1990s, including rotational atherectomy (rotablation), Excimer Laser Coronary Angioplasty (ELCA), and Directional Coronary Atherectomy (DCA) [10]. However, none of these approaches substantially improved long-term outcomes, as restenosis rates remained persistently high [12].

A pivotal breakthrough came with the development of the coronary stent—a metallic mesh scaffold designed to prevent restenosis following balloon angioplasty [13,14]. Two landmark clinical trials established the efficacy of stents compared to conventional balloon angioplasty. The North American Stent Restenosis Study (STRESS) demonstrated a lower angiographic restenosis rate (31.6% vs. 42.1%) and a lower target vessel revascularization (TVR) rate (10.2% vs. 15.4%) in the stent group compared to the balloon angioplasty group. The European Benestent Study similarly showed impressive reductions in restenosis rates (22% vs. 32%) and TVR rates (13.1% vs. 22.9%) with stent implantation. Based on these results, the Palmaz–Schatz balloon-expandable stent was approved by the U.S. Food and Drug Administration (FDA) in 1994 as the first bare metal stent (BMS) for elective use, following the approval of the Gianturco–Roubin coil stent for acute closure in 1993 [15]. A significant challenge with widespread BMS use was the occurrence of subacute stent thrombosis (SAT) and late in-stent restenosis (ISR). Although antiplatelet therapy reduced SAT rates to approximately 1%, ISR remained a clinically important problem [16,17]. As BMS use expanded to high-risk populations—including patients with small vessels, long or bifurcating lesions, and diabetes mellitus—ISR and target vessel revascularization (TVR) rates climbed to 50–60% and 30–50%, respectively [17,18]. Intracoronary brachytherapy, introduced in the late 1990s to suppress ISR, achieved moderate success but was hampered by late thrombosis, geographic mismatch, high cost, and the logistical requirement for radiation oncology involvement [19,20,21].

The true revolution in stent therapy came with the introduction of drug-eluting stents (DESs), which demonstrated remarkably low in-stent restenosis rates. This advance rapidly supplanted brachytherapy and established DES as the standard of care for coronary artery stenosis. However, the evolution of stent technology can be categorized into four generations [22,23,24,25]:

- First-generation stents (1986–2000): bare metal stents (BMSs) fabricated from stainless steel or cobalt–chromium alloys. These reduced restenosis rates relative to balloon angioplasty alone but still experienced in-stent restenosis rates of 20–40%.
- Second-generation stents (2001–2010): drug-eluting stents (DESs) coated with antiproliferative agents such as sirolimus and paclitaxel, which dramatically reduced restenosis rates. Concerns persisted, however, regarding delayed endothelialization and late stent thrombosis associated with durable polymer coatings.
- Third-generation stents (2011–present): bioresorbable vascular scaffolds (BVSs) and refined DES platforms featuring thinner struts (60–80 μm), biodegradable or biocompatible polymer coatings, and optimized drug release kinetics for reduced thrombogenicity.
- Fourth-generation stents (emerging): focus on precision-engineered stents that use additive manufacturing (AM) technologies to create patient-specific complex geometries and novel functionalities previously impossible with conventional manufacturing methods.

This review focuses specifically on drug-eluting vascular stents as a representative and clinically critical application of additive manufacturing in pharmaceutical device manufacturing. Drug-eluting stents were selected as the focal application because they integrate mechanical scaffolding with controlled pharmacological release—a combination that uniquely benefits from the geometric and compositional freedoms that 3D printing affords. The scope encompasses the full manufacturing technology landscape, advanced material portfolio, and future research directions relevant to this application. It is important to note that additive manufacturing of vascular stents is not restricted to polymeric materials; metal-based and composite systems are equally viable and clinically important, as detailed in Section 2.

Literature search methodology: This review was conducted following a systematic literature identification approach. Relevant publications were retrieved from PubMed, Scopus, Web of Science, and Google Scholar using the following keywords and their Boolean combinations: “3D printing”, “additive manufacturing”, “vascular stent”, “drug-eluting stent”, “bioengineered stent”, “biodegradable polymer”, “shape memory material”, “nanocomposite”, “4D printing”, “5D printing”, “6D printing”, “drug delivery”, “biocompatibility”, and “clinical trial”. The search was limited to publications from 2010 to 2025; seminal earlier references were included if these were historically significant. Peer-reviewed journal articles, systematic reviews, and book chapters were included. Conference abstracts and non-English language publications were excluded.

### 1.2. Evolution from Traditional to 3D-Printed Stents

#### 1.2.1. Traditional Manufacturing Methods

Stent manufacturing has evolved from basic wire coiling to modern precision engineering methods [26]. Four primary fabrication techniques are currently employed: laser cutting of metal tubes, electroforming, wire braiding, and weaving. Each method confers distinct advantages with respect to mechanical performance, biocompatibility, manufacturing efficiency, and design customization [27]. Laser cutting affords exceptional precision for intricate geometries, while wire-based techniques offer greater structural flexibility. Electroforming enables the production of ultra-thin struts. These manufacturing strategies continue to advance in parallel with developments in material science and engineering, progressively enhancing stent performance, deliverability, and clinical outcomes.

Wire-based methods include knitting, welding, and braiding, and represent some of the earliest approaches to stent fabrication. Braided stents are formed by wrapping two or more wires around a cylindrical mandrel to create a three-dimensional mesh structure. Although these stents exhibit good dimensional stability, mechanical strength in the range of 500–700 MPa, and high flexibility, they tend to foreshorten upon expansion, which complicates precise deployment [28]. Knitted stents, constructed by bending wire around pins and intertwining adjacent segments, offer exceptional flexibility but may lack adequate compression resistance. Coiled stents represent the simplest wire-based configuration and are predominantly used in non-vascular applications where retrievability and flexibility are of paramount importance. Nevertheless, their low radial strength and limited expansion capacity have prompted the development of more sophisticated stent geometries.

**Figure 1:**
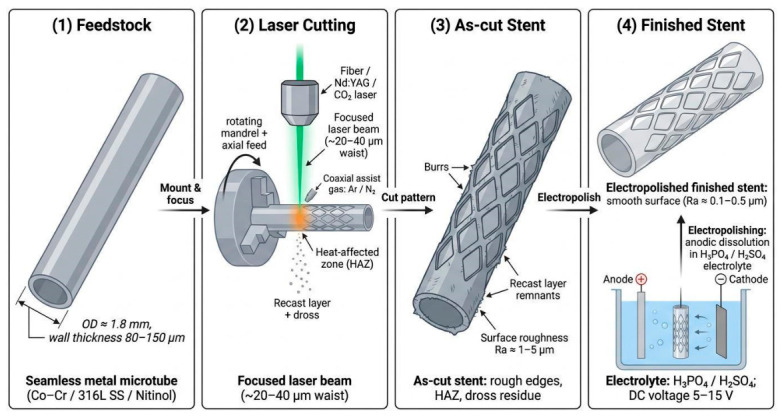
Schematic of the tube-based laser cutting process for stents. The figure should illustrate: (**1**) the seamless metal microtube feedstock, (**2**) the focused laser beam ablating the predefined strut pattern, (**3**) the cut tube with the released strut framework, and (**4**) the electropolished finished stent.

Tube-based fabrication represents the predominant approach for commercial stent production. High-energy-density lasers are widely employed to cut precise patterns into metal microtubes for the fabrication of both bare metal and polymer stents (Figure 1). Various laser types are used, including CO_2_, Nd:YAG, fiber, excimer, and ultra-short pulse lasers. Although laser cutting achieves precise material removal with minimal mechanical force, it introduces thermal effects such as heat-affected zones, striations, recast layers, microcracks, and dross formation, necessitating post-processing steps such as annealing and electropolishing [29].

Micro-electro-discharge machining (μEDM) represents another significant fabrication method. This technique uses controlled electrical pulses between a microscopic electrode and the workpiece within a dielectric fluid to create burr-free micro slots with excellent dimensional accuracy and surface smoothness [30,31]. The process can be used to fabricate stents either by patterning sheets that are subsequently folded and welded, or by directly creating patterns on cylindrical tubes. Arrays of parallel electrodes can enable efficient mass production [32].

Photochemical machining (PCM) is a flexible and cost-effective microfabrication method, also known as photochemical etching [29,33,34]. It entails coating the material with photoresist, putting a photomask on it, exposing it to ultraviolet (UV) light and chemically etching it away layer by layer [35,36]. Nevertheless, the traditional PCM does not introduce metallurgical effects, but 3D complex shape fabrication and obtaining nonuniform coatings on the tubular surfaces are challenging [36,37].

Electroforming is an electrolysis-based process in which a thin metallic layer is deposited atom by atom on a conductive substrate or mandrel until the desired thickness is reached, after which the mandrel is removed [37,38,39]. This technique allows for the precise production of complex shapes and thin-walled cylinders without seam lines using high-purity metals with controllable microstructures [40,41]. The process eliminates the need to reduce conventionally fabricated ingots through multiple working processes [42].

Hot extrusion and drawing processes are crucial for producing the tubular precursors used in stent manufacturing. Hot extrusion involves forcing heated material through a die to create solid or hollow profiles, while drawing processes apply tensile stresses to stretch hollow billets, reducing cross-sectional area while improving surface finish and dimensional accuracy [43,44,45]. For magnesium-based biodegradable stents, specialized processes like die-less mandrel drawing have been developed to achieve more significant cross-section reductions in a single step [46,47,48].

Severe plastic deformation (SPD), the assessment of microstructural refinement, now relies on severe plastic deformation (SPD) techniques which integrate cyclic extrusion and compression (CEC) with equal channel angular processing (ECAP) and double extrusion [28,49]. Bulk nanostructured and ultra-fine-grained metals with better mechanical properties arise from these techniques, which involve large plastic deformations [50]. A limited yet promising approach exists for using these techniques on stent micro-tubes.

The features of manufactured cardiac stents originate from each production process, which shapes their dimensions and internal structure and affects their resistance to corrosion and physical characteristics. A fabrication technique selection needs to consider material compatibility and desired design features and required performance standards in the vascular environment.

#### 1.2.2. Additive Manufacturing Technologies

**Figure 2:**
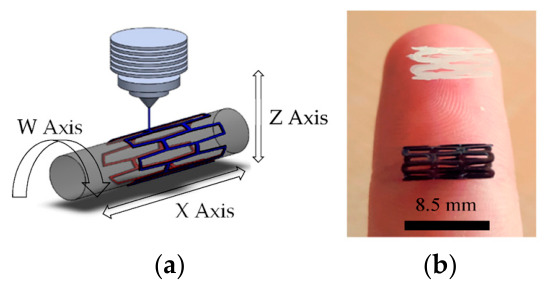
Overview of the additive manufacturing approach to vascular stent fabrication. (**a**) Schematic representation of a typical layer-by-layer 3D printing machine methodology, illustrating the deposition of material from a printing head onto a build platform along a programmable tool-path to generate a tubular stent geometry directly from a computer-aided design (CAD) model. (**b**) Representative examples of 3D-printed vascular stents produced by this approach, demonstrating the geometric complexity and design freedom achievable, including helical strut patterns, gradient porosity, and patient-specific cylindrical profiles unattainable through conventional subtractive routes. Adapted from Ref. [54].

Unlike traditional methods, additive manufacturing, which builds structures layer by layer (Figure 2), offers unprecedented design freedom in both material composition and functional integration [51]. Unlike conventional subtractive and formative manufacturing approaches (laser cutting, EDM, electroforming, wire braiding) that remove material from bulk stock or shape it through dies and molds, additive manufacturing constructs stents directly from digital models, enabling internal drug-eluting channels, gradient porosity, and complex lattice architectures that are geometrically impossible through traditional routes. This fundamental difference in manufacturing paradigm warrants a dedicated analysis presented separately from the conventional methods described in Section 1.2.1. Importantly, AM-fabricated stents are not confined to polymeric materials alone; metallic alloys (stainless steel, cobalt–chromium, nitinol) and composites are equally amenable to AM processes, as detailed in Section 2. Regarding standard dimensional requirements, coronary stents typically range from 2.25 to 5.0 mm in deployed diameter and 8 to 40 mm in length, with strut thicknesses of 60–150 μm; peripheral vascular stents are larger (5–25 mm diameter, up to 150 mm length), while neurovascular stents are considerably smaller (2–4.5 mm diameter). These targets guide the resolution and precision requirements for each AM technique discussed below. Several generations of technological advancements have occurred in the development of 3D-printed stent technology. Basic feasibility testing of the concept took place between 2010 and 2015 within the first technology generation [52]. The second-generation advancements from 2016 to 2020 brought enhanced resolution to printing and material processing that devised sophisticated designs along with bioactive procedures. Multiple functional capabilities now exist for the third-generation innovation, which operates simultaneously with bioactive materials and smart designs, alongside artificial intelligence (AI) advanced computing for individual patient planning [53].

Vat Photopolymerization:

Vat photopolymerization is among the most widely adopted 3D printing techniques for vascular stent manufacturing, particularly for polymer-based stent prototypes. This technology encompasses several variants, including stereolithography (SLA), digital light processing (DLP), and continuous liquid interface production (CLIP).

Among AM techniques, vat photopolymerization has gained prominence for polymer stent fabrication owing to its 25 μm resolution, which enables strut thicknesses approaching the 60–80 μm benchmark of conventional laser-cut stents. This resolution is well-suited to the fabrication of advanced stent architectures featuring fine structural elements and complex lattice configurations [55,56]. The process typically involves a build platform submerged in a vat of photosensitive resin that is cured layer by layer. Specialized biocompatible and bioresorbable resins have been developed for stent manufacturing, including modified polylactic acid (PLA), polycaprolactone (PCL), and various photocurable polymers with tunable mechanical properties [57]. Critical analysis of the literature, however, reveals three unresolved challenges: (1) a limited library of FDA-approved photocurable, bioresorbable resins; (2) a resolution–build-volume trade-off that restricts printable stent lengths to approximately 40 mm; and (3) a lack of long-term in vivo data beyond six months for CLIP-printed devices. These limitations confine vat photopolymerization largely to research and prototyping, and its full clinical translation requires substantial material development. Furthermore, the high equipment costs (>$150,000) and specialized resin materials restrict accessibility for small-scale laboratories and hospital-based manufacturing.

Powder Bed Fusion

Powder bed fusion (PBF) encompasses an important family of additive manufacturing processes for producing metal-based vascular stents, including selective laser melting (SLM), selective laser sintering (SLS), direct metal laser sintering (DMLS), and electron beam melting (EBM). These techniques operate by selectively melting or sintering powdered feedstock using a high-energy beam, building structures layer by layer [58]. PBF technologies are particularly advantageous for vascular stent fabrication due to their broad material compatibility and superior mechanical performance. They are predominantly applied to metallic stents using materials such as 316L stainless steel, cobalt–chromium alloys, and nitinol (NiTi), with a growing number of studies exploring polymer and composite formulations [59].

In PBF processing, a thin layer of metal powder is spread across a build platform and selectively fused using a laser or electron beam according to the prescribed design. This layer-by-layer approach enables the fabrication of intricate three-dimensional structures with feature resolutions of 20–100 μm. PBF surpasses conventional stent manufacturing in its ability to realize complex contemporary geometries that are otherwise unachievable. Finazzi et al. demonstrated the fabrication of patient-specific cardiovascular stents with 100 μm strut diameters, showing favorable mechanical properties and dimensional accuracy [60].

PBF also enables the fabrication of complex geometries—such as auxetic or functionally graded structures—that are difficult or impossible to produce by conventional methods. This design freedom facilitates the creation of stents with spatially tailored mechanical properties, for example, incorporating flexible regions to conform to tortuous anatomy and stiffer regions to maintain hoop strength. However, several important limitations must be considered. The high processing temperatures for CoCr alloys (1330–1430 °C) induce residual thermal stresses that can cause micro-cracking in thin struts (<100 μm), necessitating post-process annealing that may alter the material’s mechanical characteristics. PBF production times of 2–4 h per stent contrast unfavorably with conventional laser cutting (minutes per stent), limiting utility for high-volume or emergency manufacturing. Additionally, the as-printed surface roughness (Ra = 5–15 μm) typically requires electropolishing to achieve hemocompatible surfaces (Ra < 0.5 μm), adding processing steps and cost [61,62].

Material Extrusion

Material-extrusion-based additive manufacturing has been successfully employed primarily for polymer stent production. This category includes fused deposition modeling (FDM) and direct ink writing (DIW), both of which build three-dimensional structures by selectively depositing material through a heated or pressurized nozzle in a predefined pattern.

Material extrusion offers several advantages for vascular stent fabrication, including relatively low equipment cost, high processing speed, and compatibility with a broad range of thermoplastic polymers and composites. In FDM, a thermoplastic filament is fed through a heated nozzle that traces a computer-defined path, depositing molten material that solidifies upon cooling [63]. Polymer-based stents have been successfully produced using biocompatible, biodegradable materials including PLA, PCL, and thermoplastic polyurethanes (TPUs), which can be blended or formulated to achieve specific mechanical properties and degradation profiles appropriate to the intended clinical application [64,65].

Guerra et al. demonstrated the fabrication of biodegradable polymer stents via material extrusion, highlighting the technology’s versatility in producing diverse stent geometries [66]. Cabrera et al. investigated the fabrication mechanisms for thermoplastic copolyester (TPC) stents designed to integrate with vessel walls [67]. The relative accessibility and low cost of material extrusion systems make them particularly well-suited to rapid prototyping and iterative design optimization in research and development settings.

Material extrusion does, however, have notable limitations for vascular stent production. Standard FDM/DIW systems achieve layer heights of 200–400 μm, which precludes fabrication of struts below 200 μm—substantially exceeding the clinically preferred range of 60–140 μm that is associated with reduced restenosis risk. The resulting surface roughness also creates irregular flow patterns and increased platelet adhesion sites relative to conventionally manufactured stents, restricting material extrusion primarily to prototype development and large-diameter peripheral stents (>5 mm) where strut thickness is less critical.

#### 1.2.3. Emerging Technologies (4D, 5D, 6D Printing)

Recent developments in additive manufacturing have extended beyond conventional 3D printing to dynamic, multidimensional approaches that have the potential to transform the production of vascular stents. The emerging technologies of 4D, 5D, and 6D printing introduce additional degrees of design freedom and functional capability that significantly enhance stent performance and clinical applicability.

4D Printing: Adding Time as a Dimension

4D printing integrates the dimension of time into the additive manufacturing process by employing stimuli-responsive materials whose shape or properties change upon exposure to specific environmental triggers [68]. This capability enables the fabrication of self-deploying vascular stents that can be delivered in a compact, collapsed configuration and autonomously expand to their functional geometry upon reaching the target site [69,70]. The most clinically promising stimuli-responsive materials for 4D-printed vascular stents include thermally activated shape-memory polymers (SMPs) that recover their programmed shape at body temperature (37 °C), moisture-responsive hydrogels that react to physiological fluids, and magnetically responsive composites containing iron oxide nanoparticles [71].

Several studies have demonstrated the clinical potential of 4D-printed vascular stents. Wang et al. developed a 4D-printed peripheral vascular stent with programmable degradation behavior under fluid shear stress [69]. Similarly, Jia et al. created self-expandable vascular stents from biodegradable shape-memory polymers that activate at body temperature, eliminating the need for balloon expansion during deployment [72]. Integrating shape-memory alloys (SMAs) like nitinol with additive manufacturing has been particularly successful. Yan et al. investigated the process–microstructure–property relationships of 3D-printed nitinol stents, demonstrating superelastic behavior with full recovery from strains up to 8% and uniform.

Recent advances (2022–2025) have substantially expanded the design space for 4D-printed vascular stents and clarified their translational pathway. Gautam and Wairkar (2024) conducted a systematic review of 3D-printed bioresorbable vascular stents and concluded that polymer-based 4D platforms are now the most actively investigated route to patient-specific bioresorbable scaffolds, with PLA, PCL, and their copolymers dominating the published literature [73]. Lin et al. demonstrated that 4D-printed personalized shape-memory PLA vascular stents with negative Poisson’s ratio (auxetic) structures exhibit synchronized radial expansion without foreshortening, addressing one of the principal limitations of braided self-expandable stents [74]. Kim et al. (2024) introduced a magneto-responsive 4D-printed shape-memory nanocomposite containing iron oxide nanoparticles, enabling on-demand contactless deployment via an externally applied magnetic field and offering a new modality for remote stent activation in tortuous anatomy [75]. Shen et al. (2024) reported the first fully X-ray visible 3D-printed biodegradable vascular stent, validated by rabbit carotid artery implantation, demonstrating both reliable in vivo radiopacity and complete bioresorption within the target timeframe [76]. Rahmatabadi et al. (2024) developed sustainable shape-memory PLA–polybutylene adipate terephthalate (PLA/PBAT) blends specifically optimized for 4D printing, achieving shape-recovery ratios above 95% while improving toughness over neat PLA—an important step toward clinically translatable 4D stent feedstocks [77]. Collectively, these studies confirm that 4D printing has matured from proof-of-concept demonstrations to clinically oriented design optimization, with active programs targeting peripheral, coronary, and neurovascular indications.

5D Printing: Multi-Axis Control

The addition of multi-axis movement in fabrication constitutes a higher level of sophistication in 5D printing. The construction of 5D buildings progresses differently from conventional 3D buildings because they depend on rotational and tilting movements to create complex structures without needing supporting elements [78]. Vascular stents benefit substantially from 5D printing due to its numerous primary advantages in production. The process of removing support structures produces top-quality surface finishing and enables the creation of self-contained structures, which boosts mechanical properties and structural stability [79]. The technology allows manufacturers to precisely control fiber orientation, enabling them to create mechanical anisotropy patterns that replicate blood vessel natural properties. Using 5D printing enables the production of impossible-to-make complex geometries and overhanging features, in addition to serving other vital applications. While we are still in the early development stages of 5D printing technology for vascular applications, it seems promising to build biomimetic stents with regional mechanical properties closer to those of the heterogeneous vasculature.

- 6D Printing: Integrated Multi-Dimensional Approach

Additive manufacturing has culminated in 6D printing, which bears some traits of 4D and 5D printing. The integrated technology described here circumvents process-associated limitations in producing stimuli-responsive stents with complex geometries and multi-functionality [78,80,81]. 6D printing of vascular stents enables, among others, multi-material structures with stimuli-responsive, programmable mechanical behaviors, functionally graded properties across the entire stent structure, spatially defined drug release, spatially determined drug dose control, and the incorporation of sensing capabilities to monitor vascular conditions in situ continuously [82,83]. The new technology may produce multifunctional stents which can achieve various functions, including drug delivery, imaging, and sensing. Integration of multiple functionalities in a single device has been employed in stents, which has the potential to change the use of stent design and move towards more involved and individualized vascular disease treatment paradigms [84].

#### 1.2.4. Comparative Analysis of AM Techniques for Stent Production

This section thoroughly explored a comparison of the technical capabilities and practical considerations of primary AM technologies in vascular stent manufacturing. From the data presented in Table 1, vat photopolymerization (SLA/DLP) emerges as the most suitable platform for high-resolution polymer stents intended for drug delivery, owing to its 25–50 μm resolution, which aligns well with the fine strut dimensions (60–150 μm) required in coronary and neurovascular applications. Powder bed fusion (selective laser melting/sintering) is the preferred platform for metallic and ceramic stents where mechanical strength is paramount, though its higher cost and residual thermal stresses require careful post-processing. Material extrusion (FDM/DIW) is most applicable in early-stage prototyping and research environments where rapid iteration outweighs the need for fine surface finish. Among all platforms, vat photopolymerization and electrohydrodynamic (EHD) printing are considered the most promising for drug-eluting vascular stents specifically because they combine high geometric resolution with compatibility for drug-loaded photocurable resins that enable spatially controlled drug deposition within the stent struts. EHD printing’s sub-micron resolution is particularly attractive for next-generation multi-drug stents, although throughput limitations currently preclude clinical-scale production.

The synthesis of Table 1 reveals several clinically relevant trends that drive technology selection. First, there is a clear trade-off between resolution and throughput: vat photopolymerization and electrohydrodynamic printing achieve the finest features (25 μm and sub-micron, respectively) but at substantially reduced build rates compared with material extrusion or binder jetting, which limits their suitability for high-volume manufacturing of standard coronary stents. Second, drug-loading capability differs markedly across techniques: vat photopolymerization, material jetting, and EHD printing readily accommodate drug incorporation directly within the printed resin or droplet, whereas powder bed fusion of metals requires post-print coating because the high process temperatures (above 1000 °C) destroy most pharmaceutical agents. Third, surface finish is generally inferior in AM-fabricated stents (Ra 0.5–2.0 μm) compared with laser-cut/electropolished conventional stents (Ra 0.1–0.5 μm); this gap has direct implications for thrombogenicity and must be addressed through optimized post-processing. Fourth, only material jetting and emerging multi-material EHD platforms currently support functional gradients within a single build, which is the principal enabling capability for spatially programmed drug release across the abluminal and luminal stent surfaces. In summary, vat photopolymerization currently offers the most favorable balance of resolution, drug-loading flexibility, and process maturity for polymeric drug-eluting stents, while powder bed fusion remains the platform of choice for high-strength metallic scaffolds.

To complement the qualitative comparison of Table 1, Table 2 consolidates representative published case studies for each of the eight AM techniques discussed above, illustrating their practical implementation for vascular stent fabrication. These case studies were selected to represent both established and emerging routes, with explicit emphasis on the materials employed, stent geometry, and reported performance outcomes.

The cost structure summarized in Table 3 highlights the fundamentally different economic regimes governing traditional and additive manufacturing for stent production. The 90–99% reduction in tooling/setup cost together with the >99% reduction in customization cost reposition AM as the economically rational platform for patient-specific stents and low-volume specialty geometries, where conventional manufacturing is prohibitively expensive on a per-unit basis. Conversely, at production volumes above 10,000 units, the unit cost penalty of 3–5× for AM continues to favor laser-cut metal stents for the high-volume coronary DES market. The lead-time reduction from 3–6 months to 1–2 weeks for new designs has direct clinical implications: it allows for iterative validation cycles that are simply not compatible with traditional tooling-based workflows and enables true on-demand production for emergent cases. The 50–65% improvement in material utilization is particularly relevant when working with expensive feedstocks such as platinum–iridium or refractory alloys. Taken together, these data support a hybrid manufacturing strategy in which conventional high-volume production is retained for standard catalog stents, while AM is deployed selectively for patient-specific, complex-geometry, and small-batch indications [51,53,58].

#### 1.2.5. Performance Metrics Comparison

Quantitative analysis of mechanical and functional performance reveals distinct advantages and limitations for each manufacturing approach. Table 4 shows the performance metrics comparison between the traditional and 3D-printed of manufacturing stents.

The performance metrics in Table 4 reveal that 3D-printed stents currently match conventional stents on most safety-critical performance parameters (radial strength, fatigue life, elastic recoil) while underperforming on the strut thickness, surface roughness, and crimping profile dimensions that translate most directly to clinical outcomes such as restenosis and deliverability. The slightly thicker struts (80–200 μm for AM vs. 60–140 μm for conventional) are a direct consequence of current process resolution limits and represent a key target for next-generation AM equipment. The higher as-built surface roughness (Ra 0.5–2.0 μm vs. 0.1–0.5 μm) is clinically meaningful because it is positively correlated with platelet activation and thrombogenicity, and largely explains why current AM stents remain restricted to research and patient-specific applications rather than mainstream coronary intervention. Conversely, AM-fabricated stents demonstrate clear advantages in drug-loading capacity (up to 500 μg/cm^2^), programmable spatial drug release, and full 3D design freedom for biomimetic geometries—capabilities that cannot be replicated by conventional coating-on-laser-cut platforms. These trends reinforce the conclusion that the value proposition of AM is not as a wholesale replacement for conventional stent manufacturing but as a complementary route for high-performance, drug-engineered, and patient-specific devices.

#### 1.2.6. Clinical Outcomes Analysis

While large-scale clinical data for 3D-printed stents remains limited due to their recent introduction, early studies and preclinical data provide insights into comparative performance. Table 5 shows the clinical outcomes analysis between the traditional and 3D-printed of manufacturing stents.

The clinical data in Table 5 must be interpreted with caution because the AM-stent figures are derived largely from animal models and small first-in-human feasibility studies, whereas conventional-stent figures reflect decades of randomized trial evidence in tens of thousands of patients. With that caveat, two observations are notable. First, the projected reduction in 6-month in-stent restenosis (3–12% for AM vs. 5–15% for conventional DES) and the shortening of endothelialization time (2–4 vs. 3–6 months) are biologically plausible because they follow directly from thinner struts (proven in conventional DES to reduce restenosis) and from spatially programmed drug release achievable by AM but not by surface coating. Second, the slightly higher early-thrombosis signal for AM stents (0.8–2.0% vs. 0.5–1.5%) likely reflects the higher as-built surface roughness discussed previously and is the principal safety parameter that must improve before regulatory clearance for routine coronary use. The clear win for AM is in patient-specific fit: the ability to print stents matched to a patient’s vascular computed tomography/magnetic resonance imaging (CT/MRI) anatomy is unattainable with conventional inventory of standard sizes and is the most compelling clinical rationale for AM stent technology in complex anatomies such as ostial lesions, bifurcations, and aortic-arch peripheral disease.

#### 1.2.7. Manufacturing Efficiency Metrics

The efficiency metrics in Table 6 reinforce why AM is best positioned as a complementary rather than substitutive technology at present. The 10–50-fold lower production speed (10–50 units/day for AM vs. 500–1000 units/day for conventional lines) is the single most important factor preventing AM from replacing conventional stent manufacturing in the commodity DES segment. Conversely, the 95% reduction in design iteration time (3–6 months → 1–3 days) is exactly the metric that matters for early-stage device development, regulatory feasibility studies, and patient-specific medicine. The fivefold reduction in material waste (60–70% → 5–15%) becomes increasingly important as feedstocks transition to premium materials such as nitinol, platinum–iridium, or pharmaceutical-grade biodegradable polymers, where bulk-stock subtractive routes are economically unsustainable. The two-fold higher energy consumption for AM (100–300 vs. 50–100 kWh/kg) is a real but addressable concern that newer-generation laser sources and direct-energy-deposition systems are progressively narrowing. The clear strategic conclusion is that traditional manufacturing should remain the platform for high-volume, standardized stents, while AM is reserved for low-volume, complex, or patient-specific products where its efficiency profile is decisively favorable.

#### 1.2.8. Value Proposition Analysis

The comparative analysis reveals distinct value propositions for each manufacturing approach:

Traditional Manufacturing Advantages:

- Superior for high-volume production (>10,000 units annually) [26,51];
- Lower unit costs at scale [29,63];
- Established quality control protocols [109,113];
- Proven long-term clinical outcomes [1,9,13,17];
- Existing regulatory pathways [22,24].

3D Printing Advantages:

- Optimal for personalized/low-volume production (<1000 units) [61,108];
- Rapid prototyping and design iteration [52,66];
- Complex geometries and gradient properties [60,62,84];
- Reduced inventory and supply chain costs [58];
- Enable next-generation functionalities (4D/5D/6D printing) [69,78,82].

#### 1.2.9. Break-Even Analysis

Economic modeling indicates that 3D printing becomes cost-competitive with traditional manufacturing at production volumes below 500–1000 units annually [51,58,63], with the break-even point influenced by:

- Design complexity (lower volumes for complex designs) [62];
- Customization requirements (immediate advantage for patient-specific) [61,108];
- Material costs (closer parity for expensive alloys) [104,125];
- Regulatory requirements (additional costs for novel designs) [24,58].

This quantitative analysis demonstrates that while traditional manufacturing maintains advantages for standardized, high-volume production, additive manufacturing offers compelling benefits for personalized medicine applications, complex designs, and rapid innovation cycles. The future likely involves hybrid manufacturing strategies leveraging the strengths of both approaches to optimize clinical outcomes and economic efficiency [51,53,58].

## 2. Materials for AM Vascular Stents

The performance of vascular stents is greatly determined by selecting appropriate materials that can influence the stents’ mechanical properties, biocompatibility, degradation behavior and clinical outcomes. Additive manufacturing technologies have greatly enabled using a much wider range of materials and vascular stent designs. This section covers in-depth studies on 3D-printed vascular stent materials ranging from standard to specialty, their characterizations and the criteria for selecting particular materials for a specific clinical application.

### 2.1. Metal-Based Materials

Metal-based materials have historically dominated conventional stent manufacturing and play a significant role in 3D-printed vascular stents due to their excellent mechanical properties and established clinical track record. As evidenced by publication trends from 2012 to 2023, metals remain the most frequently utilized material category for 3D-printed vascular stents, polymers, alloys, and ceramics [128].

#### 2.1.1. Traditional Metals (Stainless Steel, Cobalt–Chromium)

Due to their well-established performance characteristics and regulatory acceptance, traditional metallic materials, mainly stainless steel and cobalt–chromium alloys, are the foundation for many 3D-printed vascular stents. Stainless steel (316L) offers tensile strength of 590–690 MPa (590–690 MPa), acceptable ductility (40–45% elongation), and good fatigue resistance [109,129,130]. It demonstrates high compatibility with powder bed fusion technologies, particularly selective laser melting (SLM) and direct metal laser sintering (DMLS). In clinical applications, stainless steel provides reliable radial strength for vessel scaffolding with established long-term outcomes. However, it exhibits higher thrombogenicity than newer alloys, relatively high density (7.9 g/cm^3^) resulting in less radiopacity, and potential for nickel ion release, causing inflammatory responses [113].

Cobalt–chromium alloys (L605, MP35N) provide a superior yield strength-to-weight ratio compared to stainless steel, radiopacity sufficient for fluoroscopic visualization without additional radio-opaque markers, exceptional corrosion resistance, and higher elastic modulus (210–235 GPa), enabling thinner strut designs while maintaining radial strength. These alloys require precise parameter optimization in powder bed fusion processes due to their high melting points (1330–1430 °C), though a surface finish of Ra < 0.5 μm after electropolishing is achievable with optimized parameters [110,112,117,131,132]. Clinically, cobalt–chromium enables thinner strut designs (as low as 65–80 μm) that promote better endothelialization and reduced restenosis rates. Finazzi et al. successfully demonstrated the production of balloon-expandable cardiovascular stents in CoCr alloy using selective laser melting with excellent mechanical properties and dimensional accuracy [104].

3D printing of traditional metals for vascular stents offers several significant benefits compared to traditional practices while developing complex shapes which enhance both blood flow optimization and material performance [107]. The layer-by-layer fabricating method enables fabricating gradient structures that contain different properties to meet various mechanical requirements in stent sections.

#### 2.1.2. Novel Metallic Alloys

Beyond traditional metals, novel alloy compositions have been developed specifically for 3D-printed vascular applications, offering enhanced functional properties and biological responses. Nitinol (nickel–titanium) exhibits remarkable superelasticity and shape-memory properties with strain recovery up to 8% compared to <1% for stainless steel [114,115,133]. Its lower elastic modulus (40–75 GPa) more closely matches native vessel properties. While challenging to process via additive manufacturing due to high reactivity with oxygen, requiring specialized inert atmosphere controls during printing, nitinol is particularly valuable for self-expanding stent designs in peripheral vessels with significant movement and tortuous anatomy. Finazzi et al. produced customized cardiovascular superelastic NiTi stents via laser powder bed fusion with 100 μm nominal strut diameter, demonstrating superelastic behavior at body temperature and tailored mechanical properties [61].

Platinum–iridium alloys provide exceptional radiopacity for improved visualization, outstanding corrosion resistance, and cell viability exceeding 90% in ISO 10993-5 cytotoxicity assays profile [125,134,135].

Refractory metal alloys (tantalum, molybdenum) exhibit excellent radiopacity, superior corrosion resistance, and high density, enabling thin-strut designs. These materials are challenging to process due to extremely high melting points (>2000 °C) and require specialized high-temperature powder bed fusion systems [136,137]. While showing potential for neurovascular and small-vessel applications where visibility and conformability are critical, these alloys remain in early-stage research, demonstrating feasibility but requiring further development for clinical translation.

Novel alloy development specifically optimized for additive manufacturing represents an active area of research. Composition–process–property relationships are being systematically investigated to achieve optimal performance characteristics for vascular applications.

#### 2.1.3. Biodegradable Metals (Magnesium, Iron-Based)

Biodegradable metallic materials represent a paradigm shift in stent design philosophy, providing temporary scaffolding during vessel healing followed by gradual resorption, thereby eliminating the permanent foreign body presence and associated long-term complications. Magnesium alloys (WE43, AZ31) exhibit relatively rapid degradation rates (2–4 months) that can be controlled through alloying with rare earth elements (Y, Nd, Ce). Their degradation products are physiologically compatible and essential nutrients [138,139,140,141]. While offering lower strength than permanent metals, they maintain adequate mechanical properties for vascular applications when properly designed, with an elastic modulus (40–45 GPa) closer to native tissue than traditional metals [142,143]. Wang et al. developed magnesium alloy micro-tubes for biodegradable vascular stents with improved mechanical properties and a corrosion rate of less than 0.1 mm/year in simulated body fluid after annealing treatment [45].

Iron-based alloys (Fe-Mn, Fe-Au, Fe-Ag) demonstrate slower degradation than magnesium (12–24+ months), with degradation rates tunable through alloying and microstructural control. These alloys provide superior mechanical strength compared to magnesium alternatives, a fatigue endurance limit exceeding 350 MPa at 10^8^ loading cycles, and good ductility, enabling crimping and expansion [144,145,146]. Successfully printed via direct metal laser sintering and binder jetting, iron-based alloys have shown promising results in recent studies. Lin et al. demonstrated in vivo degradation and endothelialization of an iron bioresorbable scaffold [122], while Huang et al. developed Fe-Au and Fe-Ag composites as candidates for biodegradable stent materials via binder jetting, demonstrating controlled degradation with acceptable biocompatibility [106].

Zinc-based alloys represent an intermediate option, showing degradation rates between magnesium and iron (6–12 months), with degradation products generally well tolerated. These alloys offer lower strength than iron but higher than magnesium, with adequate ductility for cardiovascular applications [147,148,149]. While research on additive manufacturing of zinc alloys for stents remains limited, emerging studies show promise in powder bed fusion approaches. Zinc is an essential nutrient with potential anti-atherogenic properties, though concerns about toxicity at high local concentrations require ongoing investigation. These alloys are emerging as a promising “middle ground” between rapidly-degrading magnesium and slowly-degrading iron systems [150,151,152].

Biodegradable metal stents fabricated through additive manufacturing represent a rapidly advancing field, with significant potential to address the limitations of both permanent metal stents and polymeric biodegradable alternatives. The precise control over microstructure and geometry afforded by 3D printing enables unprecedented tuning of degradation kinetics and mechanical properties to match specific clinical requirements.

### 2.2. Polymer-Based Materials

Polymer-based materials offer distinct advantages for 3D-printed vascular stents, including greater flexibility, tunable degradation profiles, and enhanced biocompatibility compared to metallic alternatives. Their lower mechanical strength, however, may limit applications to smaller vessels and less mechanically demanding anatomical sites.

#### 2.2.1. Conventional Polymers (PCL, PLA, PLGA)

Conventional biodegradable polymers represent well-established materials with extensive characterization in biomedical applications, providing a solid foundation for 3D-printed vascular stents. Polycaprolactone (PCL) is a semi-crystalline polyester with low tensile strength (20–40 MPa) but an elongation at break of 300–500% (300–500%) [105,153]. Its low glass transition temperature (−60 °C) and melting point (59–64 °C) provide good processability across multiple printing platforms, including fused deposition modeling, selective laser sintering, and solution electrospinning. Guerra & Ciurana demonstrated 3D-printed bioabsorbable PCL stents with controlled physical features. PCL undergoes slow hydrolytic degradation (2–4 years) through ester bond cleavage with minimal acid production, leading to favorable local pH maintenance [54]. While generally demonstrating favorable biocompatibility, its hydrophobic surface requires modification to enhance cell adhesion and reduce thrombogenicity. PCL is best suited for applications requiring long-term structural support with gradual transition to native tissue.

Polylactic acid (PLA) and its stereoisomers (PLLA, PDLA) exhibit higher tensile strength (45–70 MPa) but relatively brittle behavior (elongation 5–10%) that limits application in high-deformation scenarios. Their mechanical properties are highly dependent on molecular weight and crystallinity [154,155]. PLA materials demonstrate intermediate degradation rates (1–2 years for high-molecular-weight variants), with degradation occurring through hydrolysis, producing lactic acid. The potential for autocatalytic degradation in thick sections requires consideration in design. Successfully processed via vat photopolymerization, powder bed fusion, and material extrusion, PLA has shown promising results in stent applications [156,157]. Flege et al. developed a coronary PLA stent prototype using selective laser melting [158]. Sang Jin et al. demonstrated heparin coating on 3D-printed PLLA biodegradable cardiovascular stents via mild surface modification to enhance biocompatibility [121].

Poly(lactic-co-glycolic acid) (PLGA) offers tunable mechanical properties based on the lactic–glycolic acid ratio, typically demonstrating tensile strength of 40–55 MPa with moderate elongation (3–10%). Its adjustable degradation rate (1–6 months) based on copolymer ratio provides significant design flexibility, with higher glycolic acid content accelerating degradation through a bulk erosion mechanism [118,159]. Successfully implemented in stereolithography, digital light processing, and extrusion-based techniques, PLGA requires careful parameter optimization to maintain molecular weight during processing. While demonstrating established biocompatibility, its degradation products can create an acidic microenvironment, requiring buffer integration in some applications. PLGA is particularly valuable for medium-term support applications where a tailored degradation timeline is essential [160,161].

These conventional biodegradable polymers provide a spectrum of mechanical and degradation properties that can be selected and optimized for specific vascular applications. Their established regulatory history facilitates clinical translation compared to novel materials, though mechanical limitations relative to metals remain a consideration for high-stress applications.

#### 2.2.2. Shape-Memory Polymers

Shape-memory polymers (SMPs) represent an innovative class of materials that can be programmed to change shape in response to specific stimuli, offering unique capabilities for self-expanding and conformable vascular stent designs [162,163,164]. Thermally-responsive SMPs exhibit shape recovery upon exposure to body temperature, with transition temperature tunable through composition to match physiological triggers. Examples include polyurethane-based systems, crosslinked poly(ε-caprolactone), and styrene-based copolymers [165]. Jia et al. developed 3D-printed self-expandable vascular stents from biodegradable shape-memory polymers that maintained their shape at room temperature while fully activating at body temperature. These materials enable minimally invasive delivery in compact form, followed by autonomous expansion to predetermined dimensions, eliminating the need for balloon expansion. Limitations include relatively slow actuation rate, potential for incomplete deployment, and mechanical properties generally inferior to those of metallic self-expanding systems [72].

Light-responsive SMPs undergo shape changes triggered by specific wavelengths of light (typically UV or visible spectrum), enabling precise spatial and temporal control over deployment. These systems usually incorporate photosensitive moieties like azobenzene, spiropyran, or coumarin derivatives [166,167,168]. Successfully printed via vat photopolymerization techniques with careful formulation to maintain photoresponsiveness after processing, these materials show potential for controlled sequential deployment of complex stent geometries and post-implantation adjustment using transcutaneous light application [120,169]. Challenges include limited light penetration through tissue, potential phototoxicity concerns, and complex synthesis procedures [170].

Moisture/pH-responsive SMPs activate in response to physiological aqueous environment or specific pH conditions found in target tissues. Examples include modified hydrophilic polyurethanes, chitosan derivatives, and poly(acrylic acid) copolymers [171,172,173]. Successfully printed via material extrusion and vat photopolymerization, these polymers require careful environmental control during printing. They offer potential for automatic deployment upon contact with blood and targeted response to pathological pH conditions [174,175]. Considerations include deployment kinetics dependent on diffusion processes and potential premature activation during delivery, requiring protective measures.

The integration of shape-memory polymers with 3D printing technologies enables unprecedented control over stent deployment mechanics and conformability to irregular vessel geometries. Yeazel and Becker highlighted the potential of this approach for fabricating bioresorbable SMP stents with advanced functionality while noting that mechanical properties remain inferior to metallic alternatives [120]. Current research efforts focus on enhancing the mechanical resilience of these polymers while preserving their shape-memory actuation characteristics.

#### 2.2.3. Bioabsorbable Polymers

Bioabsorbable polymers specifically engineered for vascular applications provide temporary scaffolding followed by complete resorption, eliminating long-term foreign body presence while facilitating vessel healing and remodeling. Poly(glycerol sebacate) (PGS) exhibits elastomeric behavior with tunable stiffness (0.05–1.5 MPa) through crosslinking density adjustment, excellent fatigue resistance, and strain capabilities exceeding 250% [176,177,178]. It undergoes surface erosion with linear mass loss (2–6 months), generating degradation products including glycerol and sebacic acid, both endogenous compounds. Successfully processed via indirect printing approaches and emerging direct ink writing techniques, PGS requires specialized formulations for photocrosslinking in stereolithography [179,180,181]. It demonstrates minimal inflammatory reaction, supports endothelialization, and its elastomeric properties match the vessel mechanical environment. Limitations include lower mechanical strength, necessitating careful design optimization and processing challenges due to crosslinking requirements.

Poly(trimethylene carbonate) (PTMC) is a highly flexible elastomer with excellent recovery properties, low modulus (3–10 MPa), and high elongation (>400%). It undergoes surface erosion through enzymatic mechanisms, generating non-acidic degradation products that minimize inflammatory response [182,183,184]. PTMC is compatible with stereolithography through functionalization with methacrylate groups, and it has shown promising results in recent studies [185]. Ni et al. (2023) demonstrated 3D-printed peripheral vascular stents using poly(trimethylene carbonate-b-(L-lactide-ran-glycolide)) terpolymer with controlled degradation properties [186]. PTMC exhibits minimal foreign body response and favorable surface properties for blood contact, and supports cellular infiltration. Challenges include UV or thermal crosslinking requirements and relatively low stiffness, necessitating careful structural design for adequate radial support [186].

Tyrosine-derived polycarbonates offer tunable properties ranging from rigid to elastomeric based on specific chemistry, tailorable glass transition temperatures, and good ductility. They undergo controlled surface erosion (6–18 months), generating physiologically benign degradation products, including tyrosine [187,188]. These materials require specialized formulations and process parameters, successfully processed via stereolithography and powder bed fusion. They demonstrate excellent biocompatibility, support endothelialization, and elicit minimal inflammatory response [189,190,191]. Recent research has focused on developing photocrosslinkable variants designed explicitly for vat photopolymerization processes.

Poly(propylene fumarate) (PPF) is a crosslinkable unsaturated polyester with tunable mechanical properties, offering modulus ranges of 10–30 MPa depending on crosslinking density [192,193,194]. It undergoes hydrolytic degradation over 6–12 months, producing propylene glycol and fumaric acid as degradation products. PPF is compatible with vat photopolymerization techniques due to its unsaturated backbone, enabling photocrosslinking. While generally demonstrating favorable biocompatibility, its surface hydrophobicity requires modification for optimal cellular interaction [195,196,197]. PPF shows potential for gradient structures combining rigid and flexible regions in a single stent design.

The 3D printing of bioabsorbable polymers for vascular stents represents a rapidly evolving field. New material formulations specifically optimized for additive manufacturing processes are continuously being developed, enabling increasingly sophisticated degradation profiles, mechanical properties, and biological interactions tailored to specific vascular applications.

Recent work has substantially expanded the portfolio of biodegradable polymers tailored specifically to additive manufacturing of vascular stents, reflecting the rapid pace of activity in this area. Veerubhotla and Lee demonstrated fully biodegradable 3D-printed cardiovascular stent designs with tunable strut architectures and resorption profiles [108], while Gautam and Wairkar reviewed the emerging frontiers of 3D-printed bioresorbable vascular scaffolds for personalized cardiac care, emphasizing patient-specific matching of degradation rate to vessel healing [73]. Yasmin et al. (2025) systematically surveyed the application of additive manufacturing to polymeric bioresorbable stents and identified photocurable and melt-processable resin systems as the most promising routes to clinically relevant resolution [92]. Complementary studies on 4D-printed biodegradable shape-memory polymers have shown that programmable self-expansion can be combined with controlled resorption, potentially eliminating the need for balloon deployment [198]. Collectively, these recent contributions confirm that biodegradable polymer development is among the most rapidly advancing aspects of the field, with the principal remaining challenges being the limited availability of pharmaceutical-grade printable resins and the batch-to-batch reproducibility of degradation kinetics.

Table 7 consolidates the mechanical, degradation, and AM-process-compatibility properties of the principal polymer families discussed in Section 2.2.1, Section 2.2.2 and Section 2.2.3, providing a single comparative reference for stent material selection. The quantitative values were drawn from the primary references cited in the corresponding sections of this manuscript and from systematic compilations by Gautam & Wairkar (2024) [73] and Yasmin et al. (2025) [92].

### 2.3. Composite Materials

Composite materials represent an innovative approach to overcoming the limitations of single-material systems by combining the advantageous properties of multiple components. This strategy enables the development of 3D-printed vascular stents with enhanced mechanical performance, improved biocompatibility, and advanced functionalities beyond what can be achieved with homogeneous materials.

#### 2.3.1. Metal–Polymer Composites

Metal–polymer composites integrate the superior mechanical strength of metals with the favorable biological properties and flexibility of polymers, creating hybrid systems with enhanced overall performance. Metal-particle-reinforced polymers consist of polymer matrices (typically PCL, PLA, or PTMC) reinforced with metallic particles (stainless steel, titanium, or magnesium) at concentrations of 5–30% by weight [201,202,203,204]. These compositions demonstrate significant improvements in tensile strength (30–100% increase) and modulus while maintaining reasonable flexibility, with tunable properties based on particle size, loading, and distribution. Successfully implemented in material extrusion (FDM) and vat photopolymerization (SLA), these composites require careful consideration of particle sedimentation and agglomeration during processing. They exhibit differential degradation between polymer matrix and metal reinforcement, with potential for controlled release of metallic ions with therapeutic effects. Challenges include achieving homogeneous particle distribution, potential stress concentration at particle–matrix interfaces, and complex rheological behavior affecting printability [205,206].

Core–shell structures comprise a metallic core (typically stainless steel or nitinol) providing mechanical support and a polymeric shell (biodegradable or permanent) offering biocompatibility and drug delivery capabilities. Normally produced through multi-material printing or post-print coating processes, these structures combine the mechanical resilience of metals with the biological interface advantages of polymers, enabling multi-stage functional responses. Xiao et al. demonstrated direct 3D printing of thin-walled cardiovascular stents with a functional metallic coating [127]. These hybrid structures are particularly valuable for applications requiring high mechanical performance and controlled biological interactions. However, they present processing challenges in ensuring strong interfacial bonding between dissimilar materials and managing differential thermal expansion during processing.

Gradient metal–polymer systems feature continuous or stepwise transitions between metal-dominant and polymer-dominant regions, creating functional gradients along the stent structure. Implemented through multi-material printing platforms or material composition gradients within a single print material, these systems enable regionally optimized properties such as increased flexibility at ends and high radial strength in central sections. They can be designed to create varying surface properties and degradation profiles along different regions of the stent. While representing an emerging technology with promising research results, these gradient systems currently have limited clinical implementation.

These metal–polymer composite approaches represent a promising direction for overcoming the traditional dichotomy between metals’ superior mechanical properties and polymers’ favorable biological interactions, potentially offering “best of both worlds” solutions for challenging vascular applications.

#### 2.3.2. Polymer–Ceramic Composites

Polymer–ceramic composites incorporate ceramic components into polymer matrices to enhance mechanical properties, bioactivity, degradation control, and radiopacity for improved clinical performance. The combination of biodegradable polymers including PLA, PCL, PLGA with hydroxyapatite (HA) particles or whiskers between 5 and 20% achieves successful integration [103,207,208]. The combination shows higher elastic modulus (stiffness) and greater ultimate tensile strength and better maintains shape dimensions without deformation during continued stress. These biomaterials demonstrate improved bioactivity due to their ability to boost cellular binding and display potential bone formation properties near blood vessels and better surface quality for endothelial cell attachment [209,210].

Successfully processed via material extrusion and vat photopolymerization, these composites require optimization to manage increased viscosity and potential nozzle abrasion. Challenges include particle agglomeration affecting print quality, potential brittleness at higher ceramic loadings, and inconsistent dispersion affecting mechanical reliability [211,212].

Bioactive glass–polymer systems incorporate bioactive glass types 45S5 or 13–93 into biodegradable polymer matrices to different percentages ranging from 5% to 30%. The composite system allows for regulated release of Si, Ca, P ions through its structures that leads to advantageous biological reactions, as well as increased X-ray visibility and possible antimicrobial actions [213,214]. The degradation capability of these systems relies on the ability of bioactive glass to neutralize polymer degradation byproducts using its basic dissolution reaction products [215,216]. Such composites successfully function within stereolithography and extrusion-based systems due to their careful formulation development and they become beneficial materials for applications that require imaging-friendly characteristics and beneficial biological responses [217,218,219].

Tricalcium phosphate composites use a combination of β-TCP particulate-filled polymer matrices to achieve superior material properties [220,221,222]. The materials display increased compressive strength in addition to their appropriate flexibility and show better creep resistance during tests conducted under physiological conditions. Biological research shows that tricalcium phosphate releases both calcium and phosphate ions, which work to activate cell functions, promotes endothelialization, and matches the breakdown characteristics of the material [223,224]. Material extrusion and vat photopolymerization have become successful processing techniques for these composites and recent studies demonstrate their effectiveness [225]. The research of Liu et al. led to the development of tricalcium-silicate/polyetherimide composites, which can be printed as medical implants for extended clinical use. The production of these composites faces two main problems: high viscosity that complicates printing operations and possible brittleness that needs optimal component adjustments [226].

These material compositions offer combined features of polymers and ceramics to accomplish multiple necessary functionality goals. Hand surgeons benefit from the ceramic phase because it strengthens mechanical properties while releasing ions for biological signaling along with better imaging visibility and managed degradation. Processability, flexibility and structural integrity emerge from the polymer phase of the composite.

#### 2.3.3. Nanocomposites

Nanocomposites represent an advanced class of materials incorporating nanoscale reinforcements to achieve dramatic property enhancements at relatively low loading levels, enabling high-performance 3D-printed vascular stents with multifunctional capabilities [227,228]. Graphene-based nanocomposites consist of polymer matrices containing graphene, graphene oxide, or reduced graphene oxide at low concentrations (0.1–5%) [124,199]. The materials enhance tensile strength by 50–200% while maintaining low brittleness levels because of their excellent reinforcement efficiency from high aspect ratio and surface area. Functionally, they can provide electrical conductivity to sense applications and antithrombotic properties with improved thermal conductivity for better processing control [200]. The nanocomposites demonstrate promising research results when successfully applied in stereolithography and material extrusion techniques. The research by Misra et al. showcased 3D-printed multidrug-eluting stents constructed from graphene-nanoplatelet-doped biodegradable polymer composites. The interaction between cells and materials depends on surface chemistry, while antibacterial functions and controlled inflammatory response act as biological factors [119]. The development of carbon nanotube composites uses polymeric matrices that contain CNTs (carbon nanotubes) at volumes below 2% [220,229]. These materials offer exceptional reinforcement efficiency, dramatic strength and modulus improvements, enhanced fatigue resistance, and improved creep performance [230]. Functionally, they provide potential for electrical conductivity, enabling responsive behavior and enhanced visibility under specific imaging modalities. Successfully incorporated into material extrusion and vat photopolymerization systems with specialized dispersion techniques, these composites face challenges in achieving homogeneous dispersion, potential toxicity concerns requiring surface functionalization, and increased viscosity affecting printability [231,232,233].

Nanosilicate composites consist of elastomeric materials with 1–5% exfoliated clay nanoplatelets (montmorillonite, laponite) or synthetic silicates embedded in their structures. The materials prove superior mechanical strength, which, combined with stiffness and better barrier performance, minimizes water intake, slows down degradation and adds flame retardancy. Nanosilicate composites work with different printing systems while giving particular formulations rheological benefits which enhance printing performance [233,234]. The hydrolytic degradation pattern of these composites changes because they exchange ions that counteract acidic byproducts. Surface-chemistry-dependent cell interactions exist in these materials, alongside the capability for controlled drug delivery of intercalated therapeutic agents [234,235,236,237].

Metallic nanoparticle composites incorporate metallic nanoparticles (silver, gold, iron oxide) into polymer matrices at precisely controlled concentrations [238,239]. These materials offer antimicrobial activity (silver), enhanced radiopacity (gold, bismuth), magnetic responsiveness (iron oxide), and potential catalytic effects on degradation. Multiple techniques use calcium sulfate nanocomposites successfully while managing particle aggregation and settling behaviors to show cellular response patterns based on sizeovidensity and produce therapeutic outcomes through ion release mechanisms and surface effects. The composites show specific value for functional applications which need more than mechanical support, like infection control and better viewing capabilities [106,240,241,242].

Nanocomposites represent an emerging and high-potential field in vascular stent research, uniting exceptional mechanical reinforcement with functional properties and favorable biological interactions. The nanoscale reinforcements dramatically improve material properties at relatively low loading fractions while generally maintaining printability and process compatibility. However, successful clinical translation requires rigorous toxicological evaluation of nanomaterial-specific risks, including potential cytotoxicity and long-term bioaccumulation, to ensure patient safety.

### 2.4. Biomaterials and Bioresorbable Materials

Beyond conventional synthetic materials, naturally derived biomaterials and specialized bioresorbable systems offer unique advantages for 3D-printed vascular stents, including enhanced biocompatibility, physiological recognition, and tissue-mimetic properties. Natural polymers include collagen, elastin, silk fibroin, chitosan, alginate, and hyaluronic acid derivatives. These materials offer biological recognition by cellular receptors, facilitating integration, natural enzymatic degradation pathways, and intrinsic biological signals supporting healing. Successfully implemented in modified extrusion systems, inkjet printing, and specialized vat photopolymerization after methacrylation or similar modifications, these materials generally exhibit inferior mechanical properties compared to synthetic alternatives and often require crosslinking or reinforcement for adequate performance. Qiu et al. developed 3D-printed sulfated chitosan-modified bioresorbable stents for coronary artery disease with enhanced biocompatibility [123].

A decellularized extracellular matrix (dECM) comprises a complex mixture of structural and functional proteins, glycosaminoglycans, and growth factors derived from decellularized tissues. These materials preserve the natural architecture and biochemical cues of native tissues, supporting cellular infiltration and tissue-specific differentiation [243,244,245]. Successfully implemented in specialized bioprinting approaches after solubilization and rheological modification, a dECM requires reinforcement or combination with more substantial materials for adequate mechanical support in vascular applications. These materials show particular promise for hybrid approaches combining mechanical scaffolding with regenerative potential [246].

Protein-based materials include recombinant elastin-like polypeptides, resilin-like polypeptides, and engineered protein polymers. These materials feature precisely engineered amino acid sequences providing specific mechanical and biological functions, with potential for incorporating cell-binding domains and enzymatic cleavage sites [247,248]. Compatible with various bioprinting approaches after appropriate formulation development, they offer tunable mechanical properties through sequence design and programmable degradation by incorporating specific proteolytic sites. While representing an emerging technology with promising initial results, these materials currently have limited clinical implementation [249,250,251].

Cell-laden constructs combine supportive biomaterials with living cells (typically endothelial cells, smooth muscle cells, or stem cells) for direct tissue formation [252,253]. These constructs offer direct formation of living vascular tissue, accelerated endothelialization, and active remodeling and adaptation. Specialized bioprinting techniques enable the production of constructs that preserve cellular viability through low-shear and low-temperature techniques during manufacturing [254,255,256]. The constructs initially require supporting scaffolds, although they transition to matrix production by cells with time-based evolution. The processes of cell printing and subsequent manufacturing face three vital difficulties, which are sustaining cell health during fabrication, acquiring suitable mechanical characteristics during tissue formation and managing intricate regulatory procedures [257].

Biomaterials and bioresorbable technologies represent the newest frontiers in vascular stent development, enabling genuine tissue regeneration rather than mere mechanical scaffolding. By combining biological recognition systems with cell-instructive biochemical signals and physiologically harmonized degradation pathways, researchers are developing constructs that promote superior tissue integration and minimize long-term clinical complications. Current limitations in mechanical performance and processing complexity are being addressed through hybrid strategies that combine biological materials with synthetic structural components.

### 2.5. Material Selection Criteria and Decision Framework

The selection of appropriate materials for 3D-printed vascular stents requires a systematic, multi-criteria decision process that considers several interdependent factors simultaneously. A rigorous material selection framework must address mechanical performance requirements, degradation profiles, biological interactions, manufacturing process compatibility, and clinical context [58,108]. Throughout this section, it is important to distinguish between two related but distinct mechanical properties: strength refers to the maximum stress a material can sustain before failure (i.e., tensile or yield strength), whereas modulus refers to stiffness (elastic modulus). These properties are independent; a material may be strong but compliant (e.g., elastomeric polymers) or stiff but relatively weak in tension (e.g., some ceramics).

Mechanical performance constitutes the primary selection criterion. Radial strength must be sufficient to maintain vessel patency against elastic recoil and external compression, typically requiring a radial force of 300–500 mmHg for coronary applications. Flexibility must be enough to conform to vessel tortuosity without kinking or fracture during delivery and deployment, particularly critical for peripheral and neurovascular applications. Fatigue resistance demands withstanding 10^7^–10^8^ cycles of pulsatile loading without failure for permanent implants, while degradable materials must maintain adequate properties throughout the critical healing period. Elastic recoil should be minimal after expansion to maintain deployed dimensions (typically <5% requirement), and the material must allow for crimping to deliver a profile (typically 1–2 mm diameter) without damage or permanent deformation.

Degradation and stability considerations are equally important, with the degradation timeline needing to match tissue-healing and remodeling processes. Critical strength maintenance is typically required for 3–6 months for coronary applications. Surface erosion is generally preferred over bulk degradation to maintain structural integrity during resorption, and all degradation products must be biocompatible. Materials must maintain properties after exposure to sterilization processes (ethylene oxide, gamma irradiation, or e-beam), demonstrate minimal property changes during shelf storage (typically 1–2 year shelf life required for commercial viability), and resist unexpected degradation from oxidative, enzymatic, or mechanical challenges in the physiological environment.

Hemocompatibility stands as a biological performance factor which minimizes thrombogenicity while preserving blood compatibility through assessment of platelet adhesion and activation. A functional endothelial layer needs promotion for long-term patency because surface characteristics directly affect cell attachment as well as proliferation and functional capacity. Inflammatory response must be minimal to prevent excessive neointimal hyperplasia, with material chemistry and degradation products influencing macrophage polarization and foreign body response. Materials should demonstrate minimal cytotoxicity to surrounding tissues, particularly for biodegradable materials that release degradation products. For specific applications, bioactive properties might be desirable to promote healing or actively inhibit negative remodeling.

Manufacturing process compatibility involves printability considerations, with material rheological properties (viscosity, shear-thinning behavior, surface tension) affecting process reliability. Resolution capabilities must match the required feature size for stent struts (typically 50–150 μm), with material–process interactions determining achievable precision. Each material requires optimized process parameters to maintain mechanical stability where layer connection and internal flaws along with residual stresses stand as essential factors. The necessary procedures following printing should be limited to basic operations which do not require complicated cleaning methods nor support removal or surface treatments that could affect biocompatibility or maintain dimensions. The manufacturing process requires a consideration of scalability to commercial operations along with consistency between production batches and quality screening systems, which should work effectively with fabricated materials.

Manufacturing economics depends heavily on material costs because practical and regulatory factors need assessment. The approval process for known regulatory materials is shorter than that of new materials, potentially needing longer testing requirements. Standard medical device sterilization procedures must not affect the materials’ properties since sterilization compatibility remains essential. The imaging examinations under fluoroscopy, CT and MRI play a crucial role in both implant placement and post-operative assessments but require materials that meet two requirements: they must be radio-opaque and produce minimal artifacts. The sustainable production of materials requires reliable supply chains and access to products, along with minimum dependencies that proprietary materials can create, which affects supply chain continuity.

The complete decision framework begins with a rigorous identification of the critical requirements for the specific clinical application and patient population. Candidate materials should first be screened against the two most fundamental criteria—mechanical performance and biocompatibility—before proceeding to a comprehensive characterization protocol encompassing mechanical testing, degradation analysis, hemocompatibility assessment, and manufacturing feasibility evaluation. Computational tools, including finite element analysis (FEA) and degradation simulation models, should be employed to predict material behavior under physiological loading conditions prior to physical prototyping. The selection process is iterative, with prototype performance data from in vitro and preclinical studies continuously informing refinement of the material choice and manufacturing strategy.

Through this systematic evaluation, an optimal material is identified that best resolves the inherent trade-offs between competing performance requirements for the specific medical application. Continued advances in 3D printing technologies, coupled with ongoing progress in material science, are progressively enabling more versatile, patient-specific vascular stent solutions that can be tailored to individual anatomy and clinical presentation.

### 2.6. Drug-Loading Strategies in 3D-Printed Vascular Stents

A critical practical consideration for drug-eluting stents produced by additive manufacturing is the method by which the therapeutic agent is incorporated. Three principal strategies are employed. (1) Pre-mixing: the drug (e.g., sirolimus, paclitaxel, heparin) is blended directly into the polymer or composite feedstock before printing, resulting in a homogeneous drug distribution throughout the strut matrix. This is the most common approach for material extrusion (FDM/DIW) and powder bed fusion processes with polymer matrices, and produces a sustained, diffusion-controlled release profile. (2) Post-print coating: after the structural stent scaffold is fabricated (typically from a metal or mechanically robust polymer), the drug is applied as a surface coating via dip-coating, electrospray, or vapor deposition. This approach allows for independent optimization of mechanical and pharmacological properties and is widely used for metallic DES manufactured by conventional methods, and increasingly by AM. (3) Compartmentalized reservoir designs: leveraging the geometric freedom of AM, drug-loaded reservoirs or microchannels can be incorporated directly into the stent architecture during printing, enabling spatially targeted and sequential drug release (e.g., an anti-thrombotic agent from the luminal surface and an anti-proliferative agent from the abluminal surface). This third approach is unique to additive manufacturing and represents one of the most compelling advantages of 3D printing over conventional stent manufacturing. The choice of strategy depends on the drug’s thermal stability, the selected printing process, the desired release kinetics, and regulatory considerations.

## 3. Clinical Trials and Current Industrial Practice

### 3.1. Current Industrial Manufacturing Technologies for Drug-Eluting Stents

Most of the existing commercial drug-eluting stents (DESs) are made using traditional production methods. Some of the most popular are the Xience stent (Abbott Vascular), with a scaffold of cobalt–chromium (L605) that is coated with a durable fluoropolymer (PVDF-HFP) that releases everolimus; Synergy (Boston Scientific), with an ultra-thin (74 μm) scaffold of platinum–chromium (Pt-Cr) with a bioabsorbable polymer (PLGA) that releases everolimus; and the Orsiro stent (Biotronik), which features an ultra-thin (74 μm) scaffold of cobalt–chromium (L605) with a bioresorbable, everolimus-eluting polymer (PLLA). The platforms are produced in mass quantities by laser microcutting of metal microtubes, electropolished, sterilized and incorporated with drug-coated polymers. The major benefits of these traditional platforms are that they have been proven for multi-year clinical outcomes, have optimized regulatory approval processes and can be produced at a low unit cost with high volumes. The major restriction is that it is not possible to create complex internal geometries, gradient drug distribution or patient-specific geometries. Additive manufacturing overcomes all three of these shortcomings, but it has not yet matched the production quantities, regulatory approval or cost of traditional DES. A hybrid approach, combining AM as a tool to build complex scaffolds and conventional coating technologies to introduce the drug layer, could be a short-term translation route.

### 3.2. Ongoing and Completed Clinical Trials Involving 3D-Printed Vascular Stents

There are currently limited clinical data available regarding the safety and efficacy of 3D-printed vascular stents, with most of the existing evidence coming from preclinical (animal model) studies. There are several clinical programs and trials that are relevant; however, these are ongoing or have reported preliminary results. The second-generation bioabsorbable magnesium scaffold (Magmaris, Biotronik) was assessed in the DREAMS-2G trial (BIOSOLVE-II, NCT01960504) on 123 patients who were treated for de novo coronary lesions, with 12-month target lesion failure rates of 3.0%, a landmark result for biodegradable metal scaffolds and directly applicable to the AM-produced scaffolds. The results of the ABSORB III trial (NCT01751906) enabled the understanding of limitations of polymer scaffold design, as the trial was conducted in more than 2000 patients using the Abbott PLLA-based bioresorbable vascular scaffold (BVS). The current SCAFF-PAD study is assessing a scaffold peripheral PLLA in peripheral artery disease with symptoms. In relation to stents produced directly by additive manufacturing, the first-in-human feasibility studies of clinical-grade stents have recently started, but have yet to be registered as completed trials as of the time of this writing. Preliminary in vivo testing conducted by Shen et al. (2024) showed successful in vivo implantation of a fully X-ray visible 3D-printed biodegradable vascular stent in a rabbit carotid artery model, which was found to be reliable in terms of radiopacity during bioresorption period and acceptable endothelialization at 12 weeks [76]. AM-produced flow-diverter stents have been tested in the neurovascular space in preclinical models of porcine aneurysms with good results.

## 4. Limitations, Future Perspectives, and Regulatory Considerations

### 4.1. Limitations of Current Additive Manufacturing Approaches

Though it has the ability to transform, there remain a number of significant unresolved challenges for additive manufacturing of vascular stents. The ability to produce ultra-thin-strut designs < 65 μm is still a challenge in most AM processes (practical minimum feature size ≥ 50 μm for most polymer techniques and ≥80–100 μm for PBF metal processes) as these features are known to decrease restenosis in clinical studies. The surface roughness is higher than of conventional stents (Ra 0.5–2.0 μm for most AM processes vs. 0.1–0.5 μm for laser-cut and electropolished metal stents) and could have implications for thrombogenicity. Drug stability in high temperature processes (FDM, SLS) restricts the types of drugs that can be incorporated pre-print; the drugs that are sensitive to temperatures over 60–100 °C must be loaded post-print or a photocurable resin system must be used. The mechanical properties and drug release characteristics have not yet been shown to be reproducible from batch to batch, especially at the scale necessary for clinical manufacture. Lastly, the cost of clinical grade AM equipment and the lack of pharmaceutical-grade feedstock materials suitable for manufacturing of implantable devices are practical hurdles. Those same conclusions were drawn by recent systematic analyses from Yasmin et al. (2025) [92], Limón et al. (2024) [258] and Mayers et al. (2024) [259], with the three priority bottlenecks that must be overcome before clinical-scale manufacture of AM-fabricated vascular stents becomes possible being identified: feedstock standardization, in-process quality monitoring, and harmonized regulatory pathways.

### 4.2. Future Perspectives

Key advances, with multiple potential applications in the medical field, will likely develop in the future for the field, such as: (i) automated patient-specific scaffold optimization, using CT/MRI imaging data, with AI assistance; (ii) multi-material AM platforms that can print structural, drug-loaded and sacrificial support materials in a single manufacturing step, allowing for complex reservoir architecture that is difficult to achieve with conventional manufacturing processes; (iii) in-process quality monitoring of the manufactured product, based on inline optical coherence tomography and machine learning, to detect dimensional deviations during AM process—not in post-production inspection; (iv) the advance of bioprinting technologies toward cell-laden vascular stent constructs, accelerating re-endothelialization and active vascular remodeling in situ; and (v) distributed point-of-care manufacturing, where patient-specific stents are manufactured on-site at cardiac catheterization facilities, using validated digital design files.

### 4.3. Regulatory Considerations

The regulatory process for 3D-printed vascular stents is more complicated than that of traditional stents and is still somewhat undefined. The FDA has also provided guidance documents related to Technical Considerations for Additive Manufactured Medical Devices (2017 and updated 2024) that include expectations for design control, material characterization, process validation, and post-processing verification. These guidelines are, however, largely applicable to structural orthopedic implants and not fully applicable to drug/device combination products like DES. Drug-eluting AM stents must meet both the medical device regulations (Title 21 of the Code of Federal Regulations (CFR) Part 870 for cardiovascular devices) and the pharmaceutical regulations (either 21 CFR Parts 210/211 or the combination product pathways under 21 CFR Part 3) for the drug constituent. The European MDR (EU 2017/745) also stipulates conformity assessment for active-implantable Class III devices, with special annexes devoted to new manufacturing processes. Some key requirements are: ISO 10993 biocompatibility, ISO 5840 and ASTM F2606 mechanical performance, characterization of leachable and extractable AM specific residuals (e.g., unreacted photoinitiators, support material residues), accelerated aging studies for establishing shelf-life, and a dedicated section covering process validation for AM specific parameters (layer thickness, build orientation, post processing parameters). Patient-matched devices need to be designed and validated on a case-by-case basis, increasing complexity even more. Pre-Submission (Q-Submission) meetings held in the US and pre-assessment from the Notified Bodies in the EU are strongly recommended for developers of AM-fabricated drug eluting stents.

## 5. Conclusions

The convergence of additive manufacturing technologies with advanced material science has ushered in a transformative era in vascular stent development, transcending the limitations of conventional manufacturing to enable patient-specific, multifunctional, and biologically responsive devices. This comprehensive review demonstrates that 3D printing has evolved from early proof-of-concept demonstrations to a clinically viable manufacturing platform capable of producing stents with unprecedented geometric complexity, precisely tailored mechanical properties, and integrated drug-eluting and therapeutic functionalities.

The key findings and future directions of this review are summarized as follows:

- Manufacturing technology maturation: current additive manufacturing techniques achieve resolutions of 25–100 μm, enabling fabrication of strut dimensions (50–150 μm) comparable to those of conventional stents while offering superior design freedom for complex architectures, gradient structures, and multi-material integration previously unattainable through traditional methods.
- Materials innovation: the material landscape has expanded from conventional metals (316L SS, CoCr, NiTi) to encompass biodegradable metals (Mg, Fe, Zn alloys), shape-memory polymers, nanocomposites, and bio-hybrid systems, each offering distinct advantages in mechanical performance (tensile strength: 20–690 MPa), degradation profiles (2 months to 4 years), and biological interactions.
- Emerging dimensional technologies: 4D printing introduces temporal responsiveness through stimuli-responsive materials enabling self-deployment at body temperature, 5D printing provides multi-axis control eliminating support structures, and 6D printing integrates multifunctionality including drug delivery, sensing, and adaptive mechanical properties within single devices.
- Clinical translation challenges: standardization remains paramount, requiring development of specific ISO/ASTM standards for 3D-printed medical devices, accelerated degradation testing protocols correlating with in vivo performance, and comprehensive biocompatibility assessment frameworks addressing material-specific degradation products and long-term tissue responses.
- Regulatory pathway development: establishment of clear FDA/CE regulatory guidelines specific to additively manufactured cardiovascular devices, including validation protocols for patient-specific designs, quality control standards for distributed manufacturing, and post-market surveillance requirements for novel materials.
- Computational design integration: implementation of AI-driven design optimization algorithms, patient-specific finite element analysis incorporating vessel biomechanics, and predictive models for degradation kinetics and tissue remodeling to enable truly personalized stent solutions.
- Manufacturing scalability: transition from laboratory-scale production to clinical-grade manufacturing requires development of in-line quality control systems, standardized post-processing protocols, and distributed manufacturing frameworks maintaining consistent quality across production sites.
- Future research priorities: critical areas demanding investigation include development of hybrid manufacturing approaches combining additive and subtractive techniques, integration of real-time monitoring capabilities through embedded sensors, advancement of bioprinting technologies for cell-laden constructs promoting rapid endothelialization, and establishment of comprehensive databases linking design parameters to clinical outcomes.

The trajectory of 3D-printed vascular stent technology points toward a future of personalized cardiovascular interventions where stents are designed, optimized, and manufactured for individual patient anatomy and pathophysiology. Success in this endeavor requires continued interdisciplinary collaboration among material scientists, engineers, clinicians, and regulatory bodies to translate these technological capabilities into improved patient outcomes. As manufacturing technologies mature and regulatory frameworks evolve, 3D-printed stents are poised to become the standard of care, offering superior clinical performance through patient-specific design, programmable functionality, and biological integration that fundamentally redefines the treatment paradigm for vascular disease. Achieving this vision will require the research community to address the resolution, surface quality, throughput, and regulatory challenges identified in Section 4 while continuing to exploit the unique geometric and compositional freedoms that additive manufacturing provides.

## Figures and Tables

**Table 1:** Summary of advantages and limitations of manufacturing methods for vascular stents.

Manufacturing Method	Advantages	Limitations	Reference
Vat Photopolymerization	- High accuracy and precision - Availability of material selection - Fast production speed	- Requires expert operators to fabricate precise stents - Expensive equipment and materials	[55,56,57,85]
Powder bed fusion	- High accuracy and resolution - Ability to produce complex geometries - Self-expansion feature potential	- Expensive equipment - Time-consuming process	[58,59,60]
Material extrusion	- Fast production - Low cost	- Lower resolution - Surface finish limitations	[63,64,65,66,67,86]
Binder jetting	- High throughput - Low cost	- Lower resolution - Potentially reduced mechanical strength	[51,63,87]
Direct energy deposition	- High precision for metal components - Ability to repair existing stents - Excellent mechanical properties	- Limited material options - High energy consumption - Complex parameter optimization	[88,89,90]
Material jetting	- Multi-material capabilities - High precision and smooth finish - Functional gradient properties	- Limited material range - Expensive process - Mechanical property limitations	[91,92,93,94]
Electrohydrodynamic printing	- Ultra-high resolution (sub-micron) - Precise drug-loading capacity - Controlled microstructures	- Low throughput - Limited to specific materials - Technical complexity	[95,96,97,98]
Laser-induced forward transfer	- High precision for biological components - Cell-friendly process - Potential for living tissue integration	- Limited scalability - Technical complexity - Requires specialized setup	[99,100,101,102]

**Table 2:** Case studies of additive manufacturing techniques for vascular stents.

AM Technique	Reference (Year)	Material (s)	Stent Geometry/Features	Key Outcomes
Vat Photopolymerization (SLA/DLP)	van Lith et al., 2016 [52]; Ware et al., 2022 [103]	Antioxidant bioresorbable methacrylate resin; PLA/silica micro/nano-composite	High-resolution helical-strut coronary stent; 25–50 μm features	Demonstrated high-resolution clinically relevant geometry, radial strength comparable to PLLA scaffolds, and tunable degradation.
Powder Bed Fusion (SLM/SLS)	Finazzi et al., 2020/2022/2023 [60,61,104]	CoCr (L605); superelastic NiTi	Balloon-expandable and self-expanding stents; 100 μm strut diameter	Achieved dimensional accuracy ±5 μm and superelastic recovery; mechanical performance comparable to conventionally manufactured CoCr stents.
Material Extrusion (FDM/DIW)	Guerra & Ciurana, 2018 [105]; Guerra et al., 2017 [66]	PCL; PLA/PCL composite	Bioabsorbable peripheral stent; tunable strut thickness 200–400 μm	Reported process-parameter control of strut accuracy, mechanical performance suitable for low-load peripheral applications, and rapid prototyping capability.
Binder Jetting	Hong et al., 2016 [87]; Huang et al., 2016 [106]	Biodegradable Fe-Mn-Ca/Mg alloys; Fe-Au, Fe-Ag composites	Tubular bioresorbable stent prototypes with controlled porosity	Demonstrated tunable degradation kinetics and acceptable in vitro cytocompatibility; mechanical strength enhanced via post-sintering.
Direct Energy Deposition (DED)	Liu et al., 2024 [89]; Bulla et al., 2023 [90]	Laser DED and twin-wire arc DED of equiatomic NiTi	Bulk NiTi feedstock for subsequent stent fabrication; tailored phase transformation	Achieved enhanced superelastic cycling stability after aging; demonstrated feasibility of direct repair of damaged stent struts.
Material Jetting	Chokshi et al., 2025 [91]; Yasmin et al., 2025 [92]	Multi-material photopolymer; drug-loaded resin	Drug-eluting polymer stents with spatially programmed multi-drug coatings	Demonstrated multi-material/multi-color deposition for functionally graded drug release; high feature precision (16 μm) and smooth surface finish.
Electrohydrodynamic (EHD) Printing	Seo et al., 2024 [95]; Köse & Tezcan, 2024 [96]	Biodegradable PLGA; melt-electrowritten PCL	Microscale PLGA micro-patterns on 3D polymer structures; tubular MEW stents	Sub-micron resolution achieved for drug-loaded micro-patterns; high precision deposition compatible with biodegradable polymers.
Laser-Induced Forward Transfer (LIFT)	Sahu et al., 2022 [99]; Kryou et al., 2021 [101]	NiTi alloy micro-structures; drug-loaded thin films	Micro-3D-printed NiTi shape-memory features; thin-film drug delivery layers	High lateral resolution suitable for stent surface decoration; feasibility shown for cell-compatible biomolecule deposition.

**Table 3:** Cost comparison: traditional vs. 3D-printed stent manufacturing.

Cost Parameter	Traditional Manufacturing	3D Printing (AM)	Cost Differential	References
Initial Equipment Investment	$500,000–$2,000,000 (laser cutting systems)	$150,000–$800,000 (industrial AM systems)	40–70% lower for AM	[26,29,51]
Tooling/Setup Costs	$50,000–$150,000 per design	$500–$5000 per design	90–99% reduction	[54,66]
Unit Production Cost (>10,000 units)	$15–$50 per stent	$75–$200 per stent	3–5× higher for AM	[51,63,104]
Unit Production Cost (<100 units)	$500–$2000 per stent	$100–$300 per stent	50–80% lower for AM	[53,58]
Lead Time	3–6 months (new design)	1–2 weeks (new design)	85–95% reduction	[52,61]
Inventory Costs	High (batch production)	Minimal (on-demand)	70–90% reduction	[58]
Customization Cost	$100,000+ per variant	<$1000 per variant	>99% reduction	[60,62]
Material Utilization	30–40% (significant waste)	85–95% (minimal waste)	50–65% improvement	[26,51]
Post-Processing Labor	2–4 h per batch	0.5–1 h per unit	Variable by volume	[29,104]
Quality Control	$10–20 per unit	$15–30 per unit	50–100% higher for AM	[107,108]

**Table 4:** Performance metrics: traditional vs. 3D-printed stents.

Performance Parameter	Traditional Stents	3D-Printed Stents	Clinical Significance	References
Radial Strength	350–450 mmHg (CoCr)	300–420 mmHg (CoCr)	Both exceed clinical requirement (300 mmHg)	[104,109,110]
Strut Thickness	60–140 μm	80–200 μm	Thinner struts reduce restenosis	[60,111,112]
Surface Roughness (Ra)	0.1–0.5 μm	0.5–2.0 μm	Affects thrombogenicity	[29,113]
Fatigue Life	>10^8^ cycles	10^7^–10^8^ cycles	Both meet 10-year implant life	[114,115]
Elastic Recoil	2–4%	3–6%	<5% clinically acceptable	[111,114]
Foreshortening	1–3%	2–5%	Affects deployment accuracy	[28,116]
Crimping Profile	0.9–1.2 mm	1.0–1.4 mm	Impacts deliverability	[22,117]
Drug-Loading Capacity	100–200 μg/cm^2^	150–500 μg/cm^2^	Higher capacity enables multi-drug delivery	[118,119]
Degradation Control	Limited (coating only)	Programmable throughout	Enables tailored healing response	[69,120]
Design Complexity	Limited to 2.5D patterns	Full 3D capability	Enables biomimetic designs	[51,53,84]

**Table 5:** Clinical and preclinical outcomes comparison: traditional vs. 3D-printed stents.

Clinical Parameter	Traditional Stents	3D-Printed Stents	Evidence Level	References
Technical Success Rate	95–98%	92–96%	Clinical/preclinical	[1,9,13]
In-Stent Restenosis (6 months)	5–15% (DES)	3–12% (predicted)	Preclinical data	[16,17,54]
Target Lesion Revascularization	3–8% (1 year)	2–6% (projected)	Animal models	[17,121]
Endothelialization Time	3–6 months	2–4 months	Histological analysis	[121,122,123]
Thrombosis Rate	0.5–1.5%	0.8–2.0%	Early clinical data	[16,113,124]
Patient-Specific Fit	Standard sizes only	Exact anatomical match	Computational modeling	[61,108]
Procedure Time	30–60 min	25–55 min	Pilot studies	[1,107]
Fluoroscopy Time	15–25 min	12–20 min	Enhanced visibility	[125,126]
Major Adverse Cardiac Events	5–10% (1 year)	Under investigation	Ongoing trials	[13,17,24]
Quality of Life Improvement	Standardized	15–20% better (est.)	Patient-reported	[58,108]

**Table 6:** Production efficiency comparison: traditional vs. 3D-printed stents.

Efficiency Parameter	Traditional Manufacturing	3D Printing	Advantage	References
Production Speed (units/day)	500–1000	10–50	Traditional (high volume)	[26,29,51]
Design Iteration Time	3–6 months	1–3 days	AM (95% faster)	[52,66]
Minimum Batch Size	1000–10,000	1	AM (on-demand)	[54,63]
Design Complexity Impact	Exponential cost increase	Minimal impact	AM (complexity-free)	[58,62]
Multi-Material Capability	Sequential processes	Single process	AM (integrated)	[91,93,127]
Supply Chain Complexity	High (multiple vendors)	Low (raw materials)	AM (simplified)	[51,58]
Waste Generation	60–70% material waste	5–15% waste	AM (sustainable)	[48,51]
Energy Consumption	50–100 kWh/kg	100–300 kWh/kg	Traditional (efficient)	[58,59]
Labor Requirements	5–10 operators	1–3 operators	AM (automated)	[26,104]
Scalability	Excellent	Limited	Traditional (mass production)	[51,63]

**Table 7:** Key properties of polymers used in 3D-printed vascular stents.

Polymer	AM Process Compatibility	Tensile Strength (MPa)	Elongation at Break (%)	Degradation Time (Months)	Key Limitation for Stent Use
PCL [105,153]	FDM, SLS, DIW, electrospinning	20–40	300–500	24–48	Low tensile strength; hydrophobic surface limits endothelialization; slow degradation may exceed healing window.
PLA/PLLA [154,155,156,157,158]	SLA/DLP, SLM, FDM	45–70	5–10	12–24	Brittle behavior limits high-strain deployment; acidic degradation products may induce local inflammation.
PLGA [118,159,160,161]	SLA, DLP, FDM, DIW	40–55	3–10	1–6	Bulk-erosion mechanism produces acidic micro-environment; molecular-weight loss during processing requires careful parameter control.
PGS [176,177,178,179,180,181]	SLA (photocrosslinked PGS-MA), DIW	0.5–5 (elastomeric)	>250	2–6	Mechanical strength too low for unsupported radial scaffolding; requires composite reinforcement or hybrid metal–polymer design.
PTMC [182,183,184,185,186]	SLA (methacrylated PTMC), DIW	3–10	>400	12–24	Low elastic modulus necessitates careful structural design for adequate radial support; requires UV or thermal crosslinking.
Tyrosine-derived polycarbonate [187,188,189,190,191]	SLA, SLS	30–65	10–100 (tunable)	6–18	Specialized synthesis required; limited commercial availability; photocrosslinkable variants still in development.
PPF [192,193,194,195,196,197]	SLA, DLP (photocrosslinking)	10–30	5–15	6–12	Hydrophobic surface requires modification for cell adhesion; mechanical properties depend strongly on crosslink density.
Shape-Memory Polymer (general) [72,120,162,163,164,165,166,167,168,169,170,171,172,173,174,175]	SLA, FDM, DIW, 4D printing	15–60	50–400	6–36 (tunable)	Slow actuation kinetics; risk of incomplete deployment; lower radial strength than metallic self-expanding stents.
PCL/Graphene Nanocomposite [119,124,199,200]	SLA, FDM, DIW	40–90	50–250	18–36	Particle dispersion uniformity; possible long-term safety concerns around residual nanoparticles; increased melt viscosity affects printability.

## Data Availability

No new data were generated in this study.

## References

[1] Picard F., Pighi M., Marquis-Gravel G., Labinaz M., Cohen E.A., Tanguay J.F. (2022). The ongoing saga of the evolution of percutaneous coronary intervention: From balloon angioplasty to recent innovations to future prospects. Can. J. Cardiol..

[2] Canfield J., Totary-Jain H. (2018). 40 years of percutaneous coronary intervention: History and future directions. J. Pers. Med..

[3] King S.B. (1998). The development of interventional cardiology. J. Am. Coll. Cardiol..

[4] Palmaz J.C. (2004). Intravascular stents in the last and the next 10 years. J. Endovasc. Ther..

[5] Lee S.J., Liu J., Oh S.H., Soker S., Atala A., Yoo J.J. (2008). Development of a composite vascular scaffolding system that withstands physiological vascular conditions. Biomaterials.

[6] Gensini G.G. (1963). Coronary angiography. Prog. Cardiovasc. Dis..

[7] Ryan T.J. (2002). The coronary angiogram and its seminal contributions to cardiovascular medicine over five decades. Circulation.

[8] Baim D.S., Grossman W. (2006). Coronary angiography. Grossman’s Cardiac Aatheterization, Angiography, and Intervention.

[9] Htay T., Liu M.W. (2005). Drug-eluting stent: A review and update. Vasc. Health Risk Manag..

[10] Khattak S., Sharma H., Khan S.Q. (2024). Atherectomy Techniques: Rotablation, Orbital and Laser. Interv. Cardiol. Rev. Res. Resour..

[11] Tomey M.I., Kini A.S., Sharma S.K. (2014). Current status of rotational atherectomy. JACC Cardiovasc. Interv..

[12] Tran T., Brown M., Lasala J. (2008). An evidence-based approach to the use of rotational and directional coronary atherectomy in the era of drug-eluting stents: When does it make sense?. Catheter. Cardiovasc. Interv..

[13] Byrne R.A., Stone G.W., Ormiston J., Kastrati A. (2017). Coronary balloon angioplasty, stents, and scaffolds. Lancet.

[14] Raj B., Pg P., Sapa H., Shaji S.S., T S., Kp A.U., K K., Varma P. (2025). Small-Diameter Stents in Cardiovascular Applications. Chem. Biodivers..

[15] Shim W.H., Ha J.W., Cho S.Y., Park S.H., Kim H.S., Jang Y.S., Chung N., Kim S.S. (1994). Initial clinical experience of intracoronary coil (Gianturco-Roubin) stents for management of acute dissection after balloon angioplasty. Yonsei Med. J..

[16] Pleva L., Kukla P., Hlinomaz O. (2018). Treatment of coronary in-stent restenosis: A systematic review. J. Geriatr. Cardiol. JGC.

[17] Giustino G., Colombo A., Camaj A., Yasumura K., Mehran R., Stone G.W., Kini A., Sharma S.K. (2022). Coronary in-stent restenosis: JACC state-of-the-art review. J. Am. Coll. Cardiol..

[18] Mahadevan K., Cosgrove C., Strange J.W. (2021). Factors influencing stent failure in chronic total occlusion coronary intervention. Interv. Cardiol. Rev. Res. Resour..

[19] Lim Y.K., Kim D. (2021). Brachytherapy: A comprehensive review. Prog. Med. Phys..

[20] Zaorsky N.G., Davis B.J., Nguyen P.L., Showalter T.N., Hoskin P.J., Yoshioka Y., Morton G.C., Horwitz E.M. (2017). The evolution of brachytherapy for prostate cancer. Nat. Rev. Urol..

[21] Skowronek J. (2017). Current status of brachytherapy in cancer treatment–short overview. J. Contemp. Brachytherapy.

[22] Vishnu J., Manivasagam G., Mantovani D., Udduttula A., Coathup M.J., Popat K.C., Ren P.G., Prashanth K.G. (2022). Balloon expandable coronary stent materials: A systematic review focused on clinical success. Vitr. Model..

[23] Ho M.Y., Chen C.C., Wang C.Y., Chang S.H., Hsieh M.J., Lee C.H., Wu V.C.C., Hsieh I.C. (2016). The development of coronary artery stents: From bare-metal to bio-resorbable types. Metals.

[24] Mukheja Y., Sarkar A., Arora R., Pal K., Ahuja A., Vashishth A., Kuhad A., Chopra K., Jain M. (2024). Unravelling the progress and potential of drug-eluting stents and drug-coated balloons in cardiological insurgencies. Life Sci..

[25] Omar W.A., Kumbhani D.J. (2019). The current literature on bioabsorbable stents: A review. Curr. Atheroscler. Rep..

[26] Guerra A.J., Ciurana J. (2019). Stent’s Manufacturing Field: Past, Present, and Future. Angiography.

[27] Vahabli E., Mann J., Heidari B.S., Lawrence-Brown M., Norman P., Jansen S., De-Juan-Pardo E., Doyle B. (2022). The Technological Advancement to Engineer Next-Generation Stent-Grafts: Design, Material, and Fabrication Techniques. Adv. Healthc. Mater..

[28] Vanaei S., Hashemi M., Solouk A., Asghari Ilani M., Amili O., Hefzy M.S., Tang Y., Elahinia M. (2024). Manufacturing, processing, and characterization of self-expanding metallic stents: A comprehensive review. Bioengineering.

[29] Korei N., Solouk A., Nazarpak M.H., Nouri A. (2022). A review on design characteristics and fabrication methods of metallic cardiovascular stents. Mater. Today Commun..

[30] Takahata K. (2005). Batch Manufacturing Technology Based on Micro-Electro-Discharge Machining and Application to Cardiovascular Stents. Ph.D. Thesis.

[31] Takahata K. (2009). Micro-electro-discharge machining technologies for MEMS. Micro Electron. Mech. Syst..

[32] Lappin D., Mohammadi A.R., Takahata K. (2012). An experimental study of electrochemical polishing for micro-electro-discharge-machined stainless-steel stents. J. Mater. Sci. Mater. Med..

[33] Chen T.T. (1999). Electrochemical Micromachining of Microdevices from NiTi Shape Memory Alloys. Ph.D. Thesis.

[34] Saraf A.R., Sadaiah M. (2017). Photochemical machining of a novel cardiovascular stent. Mater. Manuf. Process..

[35] Allen D.M., Simpkins M., Almond H. (2010). A novel photochemical machining process for magnesium aerospace and biomedical microengineering applications. J. Micromechanics Microengineering.

[36] Saraf A.R., Yadav S.P., Sadaiah M. (2017). Precision Photochemical Machining. Micro and Precision Manufacturing.

[37] Thorat S., Lonkar V., Patil D., Sadaiah M. Some Investigations on Photochemical Machining of Cobalt-ChromiumL605 Alloy. Proceedings of the 6th International & 27th All India Manufacturing Technology, Design and Research Conference (AIMTDR-2016).

[38] Moravej M., Prima F., Fiset M., Mantovani D. (2010). Electroformed iron as new biomaterial for degradable stents: Development process and structure–properties relationship. Acta Biomater..

[39] Schuessler A., Bayer U., Siekmeyer G., Steegmueller R., Strobel M., Schuessler A. Manufacturing of stents: Optimize the stent with new manufacturing technologies. Proceedings of the 5th European Symposium of Vascular Biomaterials ESVB.

[40] Kathuria Y.P. (2006). The potential of biocompatible metallic stents and preventing restenosis. Mater. Sci. Eng. A.

[41] Moravej M., Amira S., Prima F., Rahem A., Fiset M., Mantovani D. (2011). Effect of electrodeposition current density on the microstructure and the degradation of electroformed iron for degradable stents. Mater. Sci. Eng. B.

[42] Martinez A.W., Chaikof E.L. (2011). Microfabrication and nanotechnology in stent design. Wiley Interdiscip. Rev. Nanomed. Nanobiotechnol..

[43] Guo K., Liu M., Wang J., Sun Y., Li W., Zhu S., Wang L., Guan S. (2020). Microstructure and texture evolution of fine-grained Mg-Zn-Y-Nd alloy micro-tubes for biodegradable vascular stents processed by hot extrusion and rapid cooling. J. Magnes. Alloys.

[44] Fang G., Ai W.J., Leeflang S., Duszczyk J., Zhou J. (2013). Multipass cold drawing of magnesium alloy minitubes for biodegradable vascular stents. Mater. Sci. Eng. C.

[45] Wang J., Zhou Y., Yang Z., Zhu S., Wang L., Guan S. (2018). Processing and properties of magnesium alloy micro-tubes for biodegradable vascular stents. Mater. Sci. Eng. C.

[46] Ge Q., Vedani M., Vimercati G. (2012). Extrusion of magnesium tubes for biodegradable stent precursors. Mater. Manuf. Process..

[47] Ahadi F., Azadi M., Biglari M., Bodaghi M., Khaleghian A. (2023). Evaluation of coronary stents: A review of types, materials, processing techniques, design, and problems. Heliyon.

[48] Amani S., Faraji G. (2019). Processing and properties of biodegradable magnesium microtubes for using as vascular stents: A brief review. Met. Mater. Int..

[49] Lowe T.C. (2006). Metals and alloys nanostructured by severe plastic deformation: Commercialization pathways. JOM.

[50] Faraji G., Kim H.S., Kashi H.T. (2018). Severe Plastic Deformation: Methods, Processing and Properties.

[51] Khan M.A., Khan N., Ullah M., Hamayun S., Makhmudov N.I., Safdar M., Bibi A., Wahab A., Naeem M., Hasan N. (2024). 3D printing technology and its revolutionary role in stent implementation in cardiovascular disease. Curr. Probl. Cardiol..

[52] Van Lith R., Baker E., Ware H., Yang J., Farsheed A.C., Sun C., Ameer G. (2016). 3D-printing strong high-resolution antioxidant bioresorbable vascular stents. Adv. Mater. Technol..

[53] Ullah M., Bibi A., Wahab A., Hamayun S., Rehman M.U., Khan S.U., Awan U.A., Naeem M., Saeed S., Hussain T. (2024). Shaping the future of cardiovascular disease by 3D printing applications in stent technology and its clinical outcomes. Curr. Probl. Cardiol..

[54] Guerra A.J., Cano P., Rabionet M., Puig T., Ciurana J. (2018). 3D-printed PCL/PLA composite stents: Towards a new solution to cardiovascular problems. Materials.

[55] Düzgün D.E., Nadolny K. (2018). Continuous liquid interface production (CLIP) method for rapid prototyping. J. Mech. Energy Eng..

[56] Kaijage D.J., Lee B.J. (2024). Multiphysics simulation of continuous liquid interface production (CLIP) 3D printing technology. Int. J. Precis. Eng. Manuf. Green Technol..

[57] Arif Z.U., Khalid M.Y., Noroozi R., Sadeghianmaryan A., Jalalvand M., Hossain M. (2022). Recent advances in 3D-printed polylactide and polycaprolactone-based biomaterials for tissue engineering applications. Int. J. Biol. Macromol..

[58] Garcia-Villen F., López-Zárraga F., Viseras C., Ruiz-Alonso S., Al-Hakim F., Diez-Aldama I., Saenz-del-Burgo L., Scaini D., Pedraz J.L. (2023). Three-dimensional printing as a cutting-edge, versatile and personalizable vascular stent manufacturing procedure: Toward tailor-made medical devices. Int. J. Bioprinting.

[59] Mirzaali M.J., Bobbert F.S.L., Li Y., Zadpoor A.A. (2019). Additive manufacturing of metals using powder bed-based technologies. Additive Manufacturing.

[60] Finazzi V., Berti F., Petrini L., Previtali B., Demir A.G. (2023). Additive manufacturing and post-processing of superelastic NiTi micro struts as building blocks for cardiovascular stents. Addit. Manuf..

[61] Finazzi V., Berti F., Guillory R.J., Previtali L., Previtali B., Demir A.G. (2022). Patient-specific cardiovascular superelastic NiTi stents produced by laser powder bed fusion. Procedia CIRP.

[62] Finazzi V., Demir A.G., Biffi C.A., Chiastra C., Migliavacca F., Petrini L., Previtali B. (2019). Design rules for producing cardiovascular stents by selective laser melting: Geometrical constraints and opportunities. Procedia Struct. Integr..

[63] Hua W., Shi W., Mitchell K., Raymond L., Coulter R., Zhao D., Jin Y. (2022). 3D printing of biodegradable polymer vascular stents: A review. Chin. J. Mech. Eng. Addit. Manuf. Front..

[64] Yu P., Huang S., Yang Z., Liu T., Qilin Z., Feng J., Zeng B. (2023). Biomechanical properties of a customizable TPU/PCL blended esophageal stent fabricated by 3D printing. Mater. Today Commun..

[65] Desai S.M., Sonawane R.Y., More A.P. (2023). Thermoplastic polyurethane for three-dimensional printing applications: A review. Polym. Adv. Technol..

[66] Guerra A., Roca A., De Ciurana J. (2017). A novel 3D additive manufacturing machine to biodegradable stents. Procedia Manuf..

[67] Cabrera M.S. (2016). Computational Design of Stents for Tissue-Engineered Heart Valve Replacement. Ph.D. Thesis.

[68] Rastogi P., Kandasubramanian B. (2019). Breakthrough in the printing tactics for stimuli-responsive materials: 4D printing. Chem. Eng. J..

[69] Wang X., Zhang Y., Shen P., Cheng Z., Chu C., Xue F., Bai J. (2022). Preparation of 4D printed peripheral vascular stent and its degradation behavior under fluid shear stress after deployment. Biomater. Sci..

[70] Hatami H., Almahmeed W., Kesharwani P., Sahebkar A. (2024). Exploring the potential of 3D and 4D printing in advancing stent manufacturing for cardiovascular diseases. Eur. Polym. J..

[71] Li Y., Zhang F., Liu Y., Leng J. (2020). 4D printed shape memory polymers and their structures for biomedical applications. Sci. China Technol. Sci..

[72] Jia H., Gu S.Y., Chang K. (2018). 3D printed self-expandable vascular stents from biodegradable shape memory polymer. Adv. Polym. Technol..

[73] Gautam A.J., Wairkar S. (2024). 3D-printed bioresorbable vascular stents: Emerging frontiers in personalized cardiac care. Polym. Bull..

[74] Lin C., Zhang L., Liu Y., Liu L., Leng J. (2020). 4D printing of personalized shape memory polymer vascular stents with negative Poisson’s ratio structure: A preliminary study. Sci. China Technol. Sci..

[75] Kim Y.B., Song H., Kim S., Chun H.J. (2024). 4D printing of magneto-responsive shape memory nano-composite for stents. Smart Mater. Struct..

[76] Shen Y., Tang C., Sun B., Zhang Y., Sun X., El-Newehy M., Abdulhameed M.M., Mo X., Yan R., Wu T. (2024). Development of 3D printed biodegradable, entirely X-ray visible stents for rabbit carotid artery implantation. Adv. Healthc. Mater..

[77] Rahmatabadi D., Khajepour M., Bayati A., Mirasadi K., Yousefi M.A., Shegeft A., Aberoumand M., Soltanmohammadi K., Soleyman E., Ghasemi I. (2024). Advancing sustainable shape memory polymers through 4D printing of polylactic acid-polybutylene adipate terephthalate blends. Eur. Polym. J..

[78] Rane N., Choudhary S., Rane J. (2023). 4D/5D/6D Printing Technology in the Architecture, Engineering, and Construction (AEC) Industry: Applications, Challenges, and Future Advancements. SSRN Electron. J..

[79] Perini P. (2021). 3D Bioprinting and Its Applications in Vascular Surgery: In-Vitro and In-Vivo Tests for Future 5D Personalised Nanomedicine. Ph.D. Thesis.

[80] An J., Leong K.F. (2023). Multi-material and multi-dimensional 3D printing for biomedical materials and devices. Biomed. Mater. Devices.

[81] Georgantzinos S.K., Giannopoulos G.I., Bakalis P.A. (2021). Additive manufacturing for effective smart structures: The idea of 6D printing. J. Compos. Sci..

[82] Han X., Saiding Q., Cai X., Xiao Y., Wang P., Cai Z., Gong X., Gong W., Zhang X., Cui W. (2023). Intelligent vascularized 3D/4D/5D/6D-printed tissue scaffolds. Nano-Micro Lett..

[83] Zhang T., Zhuang B., Zhang F., Yuan T., Chen Z., Yuan B., Wang S., Qu W., Ma W., Du L. (2024). Multilayer coating of a 3D-printed tracheal stent prevents tracheal stenosis. Appl. Mater. Today.

[84] Hoare D., Bussooa A., Neale S., Mirzai N., Mercer J. (2019). The future of cardiovascular stents: Bioresorbable and integrated biosensor technology. Adv. Sci..

[85] Burkhardt F., Handermann L., Rothlauf S., Gintaute A., Vach K., Spies B.C., Lüchtenborg J. (2024). Accuracy of additively manufactured and steam sterilized surgical guides by means of continuous liquid interface production, stereolithography, digital light processing, and fused filament fabrication. J. Mech. Behav. Biomed. Mater..

[86] Alexy R.D., Levi D.S. (2013). Materials and manufacturing technologies available for production of a pediatric bioabsorbable stent. BioMed Res. Int..

[87] Hong D., Chou D.T., Velikokhatnyi O.I., Roy A., Lee B., Swink I., Issaev I., Kuhn H.A., Kumta P.N. (2016). Binder-jetting 3D printing and alloy development of new biodegradable Fe-Mn-Ca/Mg alloys. Acta Biomater..

[88] Sathishkumar M., Kumar C.P., Ganesh S.S.S., Venkatesh M., Radhika N., Vignesh M., Pazhani A. (2023). Possibilities, performance and challenges of nitinol alloy fabricated by Directed Energy Deposition and Powder Bed Fusion for biomedical implants. J. Manuf. Process..

[89] Liu Q., Feng Y., Liu B., Xie Q., Zhou J., Zhang G. (2024). Regulation of microstructure, phase transformation behavior, and enhanced high superelastic cycling stability in laser direct energy deposition NiTi shape memory alloys via aging treatment. Mater. Sci. Eng. A.

[90] Bulla A., Wu K., Shen C. (2023). Influence of local thermal cycle on the lattice and microstructure evolution of twin-wire directed energy deposition-arc fabricated equiatomic NiTi alloy. Mater. Sci. Eng. A.

[91] Chokshi S., Gangatirkar R., Kandi A., DeLeonibus M., Kamel M., Chadalavada S., Gupta R., Munigala H., Tappa K., Kondor S. (2025). Medical 3D Printing Using Material Jetting: Technology Overview, Medical Applications, and Challenges. Bioengineering.

[92] Yasmin F., Vafadar A., Tolouei-Rad M. (2025). Application of additive manufacturing in the development of polymeric bioresorbable cardiovascular stents: A review. Adv. Mater. Technol..

[93] Shu Y., Ye P., Zhang J., Chang Z. (2020). Design of equipment for preparing drug-eluting stents with single-sided coating. Technol. Health Care.

[94] Sajjad R., Chauhdary S.T., Anwar M.T., Zahid A., Khosa A.A., Imran M., Sajjad M.H. (2024). A review of 4D printing–technologies, shape shifting, smart polymer based materials, and biomedical applications. Adv. Ind. Eng. Polym. Res..

[95] Seo I., Hassan R.U., Ryu B., Koh W.G., Ryu W. (2024). Electrohydrodynamic Printing of Biodegradable PLGA Micro-Patterns on 3D Polymer Structures. Adv. Mater. Technol..

[96] Köse F., Tezcan A. (2024). Redesign and Fabrication of Stent Designs Produced by Common Methods by Optimizing For Melt Electro Writing (Mew) Method. Int. J. 3D Print. Technol. Digit. Ind..

[97] Zhang F., Cao K., Zaeri A., Zgeib R., Chang R.C. (2024). The Design and Fabrication of Engineered Tubular Tissue Constructs Enabled by Electrohydrodynamic Fabrication Techniques: A Review. Macromol. Mater. Eng..

[98] Cai S., Sun Y., Wang Z., Yang W., Li X., Yu H. (2021). Mechanisms, influencing factors, and applications of electrohydrodynamic jet printing. Nanotechnol. Rev..

[99] Sahu A., Palani I.A., Singh V. (2022). Parametric investigations on laser-induced forward transfer based micro-3D printing of NiTi alloy. Mater. Manuf. Process..

[100] Muniraj L., Ardron M., Reuben R.L., Hand D.P. (2023). Laser-Induced Forward Transfer of Ni-rich NiTi Alloys for Shape Memory Applications. J. Laser Micro/Nanoeng..

[101] Kryou C., Orfanou I.M., Kalaitzis A., Chandrinou C., Tamvakopoulos K., Zergioti I. (2021). Fabrication of thin films as a drug delivery tool via laser-induced forward transfer. Laser-Based Micro-and Nanoprocessing XV.

[102] Demir A.G., Crimella D., Piovera C. (2024). Laser induced forward transfer with high resolution for microelectronics applications. Laser Applications in Microelectronic and Optoelectronic Manufacturing (LAMOM) XXIX.

[103] Ware H.O.T., Ding Y., Collins C., Akar B., Akbari N., Wang H., Duan C., Ameer G., Sun C. (2022). In situ formation of micro/nano phase composite for 3D printing clinically relevant bioresorbable stents. Mater. Today Chem..

[104] Finazzi V., Demir A.G., Biffi C.A., Migliavacca F., Petrini L., Previtali B. (2020). Design and functional testing of a novel balloon-expandable cardiovascular stent in CoCr alloy produced by selective laser melting. J. Manuf. Process..

[105] Guerra A.J., Ciurana J. (2018). 3D-printed bioabsordable polycaprolactone stent: The effect of process parameters on its physical features. Mater. Des..

[106] Huang T., Cheng J., Bian D., Zheng Y. (2016). Fe–Au and Fe–Ag composites as candidates for biodegradable stent materials. J. Biomed. Mater. Res. Part B Appl. Biomater..

[107] Ni D.J., Yang Q.F., Nie L., Xu J., He S.Z., Yao J. (2024). The past, present, and future of endoscopic management for biliary strictures: Technological innovations and stent advancements. Front. Med..

[108] Veerubhotla K., Lee C.H. (2022). Design of biodegradable 3D-printed cardiovascular stent. Bioprinting.

[109] Raval A., Choubey A., Engineer C., Kothwala D. (2004). Development and assessment of 316LVM cardiovascular stents. Mater. Sci. Eng. A.

[110] Phan T., Jones J.E., Chen M., Bowles D.K., Fay W.P., Yu Q. (2022). A biocompatibility study of plasma nanocoatings onto cobalt chromium L605 alloy for cardiovascular stent applications. Materials.

[111] Yan L., Soh S.L., Wang N., Ma Q., Lu W.F., Dheen S.T., Kumar A.S., Fuh J.Y.H. (2022). Evaluation and characterization of nitinol stents produced by selective laser melting with various process parameters. Prog. Addit. Manuf..

[112] Sudheer S.K., Kothwala D., Prathibha S., Engineer C., Raval A., Kotadia H. (2008). Laser microfabrication of L605 cobalt-chromium cardiovascular stent implants with modulated pulsed Nd: YAG laser. J. Micro/Nanolithography MEMS MOEMS.

[113] Karanasiou G., Tachos N.S., Sakellarios A., Michalis L.K., Conway C., Edelman E.R., Fotiadis D.I. (2017). In silico assessment of the effects of material on stent deployment. Proceedings of the 2017 IEEE 17th International Conference on Bioinformatics and Bioengineering (BIBE).

[114] Kapoor D. (2017). Nitinol for medical applications: A brief introduction to the properties and processing of nickel titanium shape memory alloys and their use in stents. Johns. Matthey Technol. Rev..

[115] Nagaraja S., Chandrasekar V., Ormonde D., Hickey H., Lipschultz K., Chao C., Vilendrer K., Pelton A.R. (2018). The impact of fatigue testing and surface processing on nickel release in nitinol stents. Shape Mem. Superelasticity.

[116] Moeri L., Lichtenberg M., Gnanapiragasam S., Barco S., Sebastian T. (2021). Braided or laser-cut self-expanding nitinol stents for the common femoral vein in patients with post-thrombotic syndrome. J. Vasc. Surg. Venous Lymphat. Disord..

[117] Wang H., Wang X., Qian H., Lou D., Song M., Zhao X. (2021). The optimal structural analysis of cobalt-chromium alloy (L-605) coronary stents. Comput. Methods Biomech. Biomed. Eng..

[118] Zhu X., Braatz R.D. (2015). A mechanistic model for drug release in PLGA biodegradable stent coatings coupled with polymer degradation and erosion. J. Biomed. Mater. Res. Part A.

[119] Misra S.K., Ostadhossein F., Babu R., Kus J., Tankasala D., Sutrisno A., Walsh K.A., Bromfield C.R., Pan D. (2017). 3D-printed multidrug-eluting stent from graphene-nanoplatelet-doped biodegradable polymer composite. Adv. Healthc. Mater..

[120] Yeazel T.R., Becker M.L. (2020). Advancing toward 3D printing of bioresorbable shape memory polymer stents. Biomacromolecules.

[121] Lee S.J., Jo H.H., Lim K.S., Lim D., Lee S., Lee J.H., Kim W.D., Jeong M.H., Lim J.Y., Kwon I.K. (2019). Heparin coating on 3D printed poly (l-lactic acid) biodegradable cardiovascular stent via mild surface modification approach for coronary artery implantation. Chem. Eng. J..

[122] Lin W., Zhang H., Zhang W., Qi H., Zhang G., Qian J., Li X., Qin L., Li H., Wang X. (2021). In vivo degradation and endothelialization of an iron bioresorbable scaffold. Bioact. Mater..

[123] Qiu T., Jiang W., Yan P., Jiao L., Wang X. (2020). Development of 3D-printed sulfated chitosan modified bioresorbable stents for coronary artery disease. Front. Bioeng. Biotechnol..

[124] Yang M.C., Tsou H.M., Hsiao Y.S., Cheng Y.W., Liu C.C., Huang L.Y., Peng X.Y., Liu T.Y., Yung M.C., Hsu C.C. (2019). Electrochemical polymerization of PEDOT–graphene oxide–heparin composite coating for anti-fouling and anti-clotting of cardiovascular stents. Polymers.

[125] Bhargava B., De Scheerder I., Ping Q.B., Yanming H., Chan R., Soo Kim H., Kollum M., Cottin Y., Leon M.B. (2000). A novel platinum-iridium, potentially gamma radioactive stent: Evaluation in a porcine model. Catheter. Cardiovasc. Interv..

[126] O’Brien B.J., Stinson J.S., Larsen S.R., Eppihimer M.J., Carroll W.M. (2010). A platinum–chromium steel for cardiovascular stents. Biomaterials.

[127] Xiao R., Feng X., Liu W., Zhou W., Li X., Song I., Ding M., Pu Y., Zhang D., Fan R. (2023). Direct 3D printing of thin-walled cardiovascular stents with negative poisson’s ratio (NPR) structure and functional metallic coating. Compos. Struct..

[128] Aldawood F.K. (2023). A comprehensive review of 4D printing: State of the arts, opportunities, and challenges. Actuators.

[129] Shih C.C., Shih C.M., Chou K.Y., Lin S.J., Su Y.Y. (2007). Stability of passivated 316L stainless steel oxide films for cardiovascular stents. J. Biomed. Mater. Res. Part A.

[130] Wu T., Chen X., Fan D., Pang X. (2015). Development and application of metal materials in terms of vascular stents. Bio-Med. Mater. Eng..

[131] Poncin P., Millet C., Chevy J., Proft J.L. Comparing and optimizing Co-Cr tubing for stent applications. Proceedings of the Materials and Processes for Medical Devices Conference.

[132] Milleret V., Ziogas A., Buzzi S., Heuberger R., Zucker A., Ehrbar M. (2015). Effect of oxide layer modification of CoCr stent alloys on blood activation and endothelial behavior. J. Biomed. Mater. Res. Part B Appl. Biomater..

[133] Sharma N., Raj T., Jangra K. (2015). Applications of nickel-titanium alloy. J. Eng. Technol..

[134] Muhammad N., Whitehead D., Boor A., Oppenlander W., Liu Z., Li L. (2012). Picosecond laser micromachining of nitinol and platinum–iridium alloy for coronary stent applications. Appl. Phys. A.

[135] Woodward B.K. (2014). Platinum group metals (PGMs) for permanent implantable electronic devices. Precious Metals for Biomedical Applications.

[136] Hanawa T. (2009). Materials for metallic stents. J. Artif. Organs.

[137] Ribeiro A.M., Flores-Sahagun T.H., Paredes R.C. (2016). A perspective on molybdenum biocompatibility and antimicrobial activity for applications in implants. J. Mater. Sci..

[138] Pang T.Y., Kwok J.S., Nguyen C.T., Fox K. (2021). Evaluating magnesium alloy WE43 for bioresorbable coronary stent applications. MRS Adv..

[139] Acheson J., Fullen N., Xu Z., Roy A., McKillop S., Lemoine P., Boyd A., Meenan B.J. Tailored Degradation and Improved Biocompatibility of Magnesium Alloys for Resorbable Bone Fixation Products. Proceedings of the 10th Annual Symposium on Biodegradable Metals.

[140] Tiasha T.R. (2017). Biodegradable Magnesium Implants for Medical Applications. Master’s Thesis.

[141] Liu Y., Lu B., Cai Z. (2019). Recent Progress on Mg-and Zn-Based Alloys for Biodegradable Vascular Stent Applications. J. Nanomater..

[142] Ma J., Zhao N., Betts L., Zhu D. (2016). Bio-adaption between magnesium alloy stent and the blood vessel: A review. J. Mater. Sci. Technol..

[143] Liu K.P., Cheng A.Y., You J.L., Chang Y.H., Tseng C.C., Ger M.D. (2025). Biocompatibility and corrosion resistance of drug coatings with different polymers for magnesium alloy cardiovascular stents. Colloids Surf. B Biointerfaces.

[144] Gąsior G., Szczepański J., Radtke A. (2021). Biodegradable iron-based materials-what was done and what more can be done?. Materials.

[145] Zivic F., Grujovic N., Pellicer E., Sort J., Mitrovic S., Adamovic D., Vulovic M. (2018). Biodegradable metals as biomaterials for clinical Practice: Iron-based materials. Biomaterials in Clinical Practice: Advances in Clinical Research and Medical Devices.

[146] Sikora-Jasinska M. (2018). Design, Development and Validation of Iron-Based Composites for Biodegradable Implant Applications. Ph.D. Thesis.

[147] Bowen P.K., Shearier E.R., Zhao S., Guillory R.J., Zhao F., Goldman J., Drelich J.W. (2016). Biodegradable metals for cardiovascular stents: From clinical concerns to recent Zn-Alloys. Adv. Healthc. Mater..

[148] Mostaed E., Sikora-Jasinska M., Mostaed A., Loffredo S., Demir A.G., Previtali B., Mantovani D., Beanland R., Vedani M. (2016). Novel Zn-based alloys for biodegradable stent applications: Design, development and in vitro degradation. J. Mech. Behav. Biomed. Mater..

[149] Lin S., Ran X., Yan X., Yan W., Wang Q., Yin T., Zhou J.G., Hu T., Wang G. (2019). Corrosion behavior and biocompatibility evaluation of a novel zinc-based alloy stent in rabbit carotid artery model. J. Biomed. Mater. Res. Part B Appl. Biomater..

[150] Fu J., Su Y., Qin Y.X., Zheng Y., Wang Y., Zhu D. (2020). Evolution of metallic cardiovascular stent materials: A comparative study among stainless steel, magnesium and zinc. Biomaterials.

[151] Amano H., Miyake K., Hinoki A., Yokota K., Kinoshita F., Nakazawa A., Tanaka Y., Seto Y., Uchida H. (2020). Novel zinc alloys for biodegradable surgical staples. World J. Clin. Cases.

[152] Mostaed E., Sikora-Jasinska M., Drelich J.W., Vedani M. (2018). Zinc-based alloys for degradable vascular stent applications. Acta Biomater..

[153] Singh J., Pandey P.M., Kaur T., Singh N. (2021). A comparative analysis of solvent cast 3D printed carbonyl iron powder reinforced polycaprolactone polymeric stents for intravascular applications. J. Biomed. Mater. Res. Part B Appl. Biomater..

[154] Jumat M.A., Vangetaraman K., Shafi A.A., Zain N.M., Saidin S. (2023). Biodegradable Polymeric Stent: Poly (lactic acid) Variation. J. Hum. Centered Technol..

[155] Wang Y., Wu H., Fan S., Wu J., Yang S. (2023). Structure design and mechanical performance analysis of three kinds of bioresorbable poly-lactic acid (PLA) stents. Comput. Methods Biomech. Biomed. Eng..

[156] Lopez-Pelaez C.M. (2022). PLA Stéréo-Complexes à Blocs: De la Synthèse aux Applications Biomédicales. Doctoral Dissertation.

[157] Talja M., Välimaa T., Tammela T., Petas A., Törmälä P. (1997). Bioabsorbable and biodegradable stents in urology. J. Endourol..

[158] Flege C., Vogt F., Höges S., Jauer L., Borinski M., Schulte V.A., Hoffmann R., Poprawe R., Meiners W., Jobmann M. (2013). Development and characterization of a coronary polylactic acid stent prototype generated by selective laser melting. J. Mater. Sci. Mater. Med..

[159] Xi T., Gao R., Xu B., Chen L., Luo T., Liu J., Wei Y., Zhong S. (2010). In vitro and in vivo changes to PLGA/sirolimus coating on drug eluting stents. Biomaterials.

[160] Zhu X., Braatz R.D. (2014). Modeling and analysis of drug-eluting stents with biodegradable PLGA coating: Consequences on intravascular drug delivery. J. Biomech. Eng..

[161] Jia Z., Ma C., Zhang H. (2021). PLGA coatings and PLGA drug-loading coatings for cardiac stent samples: Degradation characteristics and blood compatibility. Coatings.

[162] Meng H., Li G. (2013). A review of stimuli-responsive shape memory polymer composites. Polymer.

[163] Leng J., Lan X., Liu Y., Du S. (2011). Shape-memory polymers and their composites: Stimulus methods and applications. Prog. Mater. Sci..

[164] Xia Y., He Y., Zhang F., Liu Y., Leng J. (2021). A review of shape memory polymers and composites: Mechanisms, materials, and applications. Adv. Mater..

[165] Pretsch T. (2010). Review on the functional determinants and durability of shape memory polymers. Polymers.

[166] Wang Y., Wang Y., Wei Q., Zhang J. (2022). Light-responsive shape memory polymer composites. Eur. Polym. J..

[167] Fang L., Yan W., Chen S., Duan Q., Herath M., Epaarachchi J., Liu Y., Lu C. (2023). Light and shape-memory polymers: Characterization, preparation, stimulation, and application. Macromol. Mater. Eng..

[168] Wang Y., Wang Y., Wang X., Mushtaq R.T., Li C., Wei Q. (2024). Infrared light responsive shape memory tracheal stent based on TPU6/PCL4/PANI composites. J. Reinf. Plast. Compos..

[169] Chu C., Xiang Z., Wang J., Xie H., Xiang T., Zhou S. (2020). A near-infrared light-triggered shape-memory polymer for long-time fluorescence imaging in deep tissues. J. Mater. Chem. B.

[170] Delaey J., Dubruel P., Van Vlierberghe S. (2020). Shape-memory polymers for biomedical applications. Adv. Funct. Mater..

[171] Li Y., Chen H., Liu D., Wang W., Liu Y., Zhou S. (2015). pH-responsive shape memory poly (ethylene glycol)–poly (ε-caprolactone)-based polyurethane/cellulose nanocrystals nanocomposite. ACS Appl. Mater. Interfaces.

[172] Zhang K., Liang W., Chen X.B., Mang J. (2025). Smart Materials Strategy for Vascular Challenges Targeting In-Stent Restenosis: A Critical Review. Regen. Biomater..

[173] Xiao R., Huang W.M. (2020). Heating/solvent responsive shape-memory polymers for implant biomedical devices in minimally invasive surgery: Current status and challenge. Macromol. Biosci..

[174] Wu H., Yang S., Li J., Ma T., Yang K., Liao T., Feng W., Zhou B., Yong X., Zhou K. (2024). Current status and challenges of shape memory scaffolds in biomedical applications. MedComm–Biomater. Appl..

[175] Zeng C., Liu L., Zhao W., Xin X., Liu Y., Leng J. (2020). Advances in Shape-Memory Polymers and Composites for Biomedical Device Applications. Adv. Eng. Mater..

[176] Kerativitayanan P., Gaharwar A.K. (2015). Elastomeric and mechanically stiff nanocomposites from poly (glycerol sebacate) and bioactive nanosilicates. Acta Biomater..

[177] Patel A., Gaharwar A.K., Iviglia G., Zhang H., Mukundan S., Mihaila S.M., Demarchi D., Khademhosseini A. (2013). Highly elastomeric poly (glycerol sebacate)-co-poly (ethylene glycol) amphiphilic block copolymers. Biomaterials.

[178] Chen Q.Z., Bismarck A., Hansen U., Junaid S., Tran M.Q., Harding S.E., Ali N.N., Boccaccini A.R. (2008). Characterisation of a soft elastomer poly (glycerol sebacate) designed to match the mechanical properties of myocardial tissue. Biomaterials.

[179] Liang S.L., Cook W.D., Thouas G.A., Chen Q.Z. (2010). The mechanical characteristics and in vitro biocompatibility of poly (glycerol sebacate)-Bioglass^®^ elastomeric composites. Biomaterials.

[180] Zaky S.H., Lee K.W., Gao J., Jensen A., Verdelis K., Wang Y., Almarza A.J., Sfeir C. (2017). Poly (glycerol sebacate) elastomer supports bone regeneration by its mechanical properties being closer to osteoid tissue rather than to mature bone. Acta Biomater..

[181] Rai R., Tallawi M., Grigore A., Boccaccini A.R. (2012). Synthesis, properties and biomedical applications of poly (glycerol sebacate)(PGS): A review. Prog. Polym. Sci..

[182] Liu X., Liu S., Feng S., Wang X., Bai W., Xiao J., Chen D., Xiong C., Zhang L. (2021). Thermal, mechanical and degradation properties of flexible poly (1, 3-trimethylene carbonate)/poly (L-lactide-co-ε-caprolactone) blends. J. Polym. Res..

[183] Song Y., Kamphuis M.M.J., Zhang Z., Sterk L.T., Vermes I., Poot A.A., Feijen J., Grijpma D.W. (2010). Flexible and elastic porous poly (trimethylene carbonate) structures for use in vascular tissue engineering. Acta Biomater..

[184] Schüller-Ravoo S., Feijen J., Grijpma D.W. (2012). Flexible, elastic and tear-resistant networks prepared by photo-crosslinking poly (trimethylene carbonate) macromers. Acta Biomater..

[185] Fukushima K. (2016). Poly (trimethylene carbonate)-based polymers engineered for biodegradable functional biomaterials. Biomater. Sci..

[186] Ni N., Fan T., Ye W., Xia Q., Liu D., Qin J., Fan Z., Liu Q. (2023). 3D printed peripheral vascular stents based on degradable poly (trimethylene carbonate-b-(L-lactide-ran-glycolide)) terpolymer. Polym. Adv. Technol..

[187] Jain S., John A., George C.E., Johnson R.P. (2023). Tyrosine-derived polymers as potential biomaterials: Synthesis strategies, properties, and applications. Biomacromolecules.

[188] Bourke S.L., Kohn J. (2003). Polymers derived from the amino acid L-tyrosine: Polycarbonates, polyarylates and copolymers with poly (ethylene glycol). Adv. Drug Deliv. Rev..

[189] Lewitus D., Vogelstein R.J., Zhen G., Choi Y.S., Kohn J., Harshbarger S., Jia X. (2010). Designing tyrosine-derived polycarbonate polymers for biodegradable regenerative type neural interface capable of neural recording. IEEE Trans. Neural Syst. Rehabil. Eng..

[190] Shah P.N. (2009). Biocompatibility Analysis and Biomedical Device Development Using Novel L-Tyrosine Based Polymers. Ph.D. Thesis.

[191] Gunatillake P.A., Adhikari R. (2003). Biodegradable synthetic polymers for tissue engineering. Eur. Cells Mater..

[192] Cai Z., Wan Y., Becker M.L., Long Y.Z., Dean D. (2019). Poly (propylene fumarate)-based materials: Synthesis, functionalization, properties, device fabrication and biomedical applications. Biomaterials.

[193] Luo Y. (2019). Synthesis, Characterization and 3D Printing of Linear and Star-Shaped Poly (Propylene Fumarate) for Medical Applications. Doctoral Dissertation.

[194] Spasojević P., Savković M.S. (2023). Potential of unsaturated polyesters in biomedicine and tissue engineering. Applications of Unsaturated Polyester Resins.

[195] Fisher J.P., Dean D., Mikos A.G. (2002). Photocrosslinking characteristics and mechanical properties of diethyl fumarate/poly (propylene fumarate) biomaterials. Biomaterials.

[196] Wang K., Cai L., Hao F., Xu X., Cui M., Wang S. (2010). Distinct cell responses to substrates consisting of poly (ε-caprolactone) and poly (propylene fumarate) in the presence or absence of cross-links. Biomacromolecules.

[197] Chen Y. (2018). Poly (Propylene Fumarate) Functionalization via Monomer Modification and Synthesis of Multifunctional Polymer. Master’s Thesis.

[198] Slavkovic V., Palic N., Milenkovic S., Zivic F., Grujovic N. (2023). Thermo-mechanical characterization of 4d-printed biodegradable shape-memory scaffolds using four-axis 3D-printing system. Materials.

[199] Duarah R., Singh Y.P., Gupta P., Mandal B.B., Karak N. (2016). High performance bio-based hyperbranched polyurethane/carbon dot-silver nanocomposite: A rapid self-expandable stent. Biofabrication.

[200] Ovcharenko E.A., Seifalian A., Rezvova M.A., Klyshnikov K.Y., Glushkova T.V., Akenteva T.N., Antonova L.V., Velikanova E.A., Chernonosova V.S., Shevelev G.Y. (2020). A new nanocomposite copolymer based on functionalised graphene oxide for development of heart valves. Sci. Rep..

[201] Mozafari H. (2019). Bioresorbable Composite Stents for Enhanced Response of Vascular Smooth Muscle Cells. Ph.D. Thesis.

[202] Jiang D., Ning F., Wang Y. (2021). Additive manufacturing of biodegradable iron-based particle reinforced polylactic acid composite scaffolds for tissue engineering. J. Mater. Process. Technol..

[203] Egbo M.K. (2021). A fundamental review on composite materials and some of their applications in biomedical engineering. J. King Saud. Univ. Eng. Sci..

[204] Bhuvaneswari V., Arulmurugan B., Balaji D., Aravindh M., Rajeshkumar L. (2024). An overview of stress analysis of composites through computational modelling and simulation with the aid of patent landscape analysis. Arch. Comput. Methods Eng..

[205] Mozafari H., Dong P., Ren K., Han X., Gu L. (2019). Micromechanical analysis of bioresorbable PLLA/Mg composites coated with MgO: Effects of particle weight fraction, particle/matrix interface bonding strength and interphase. Compos. Part B Eng..

[206] Nagabushanam M., Devade K., Reddy G.A., Goud B.N., Sayed R.M., Sood S., Sonia P. (2023). Advance biomedical engineering–A fundamental review of composite materials and its applications. Mater. Today Proc..

[207] Alizadeh-Osgouei M., Li Y., Wen C. (2019). A comprehensive review of biodegradable synthetic polymer-ceramic composites and their manufacture for biomedical applications. Bioact. Mater..

[208] Ware H.O.T., Farsheed A.C., Akar B., Duan C., Chen X., Ameer G., Sun C. (2018). High-speed on-demand 3D printed bioresorbable vascular scaffolds. Mater. Today Chem..

[209] Jain C., Surabhi P., Marathe K. (2023). Critical review on the developments in polymer composite materials for biomedical implants. J. Biomater. Sci. Polym. Ed..

[210] Singh J., Singh G., Pandey P.M. (2021). Multi-objective optimization of solvent cast 3D printing process parameters for fabrication of biodegradable composite stents. Int. J. Adv. Manuf. Technol..

[211] Nayak V.V., Sanjairaj V., Behera R.K., Smay J.E., Gupta N., Coelho P.G., Witek L. (2024). Direct inkjet writing of polylactic acid/β-tricalcium phosphate composites for bone tissue regeneration: A proof-of-concept study. J. Biomed. Mater. Res. Part B Appl. Biomater..

[212] Walke W., Paszenda Z., Marciniak J. (2005). Badania odporności korozyjnej stopu Co-Cr-W-Ni z przeznaczeniem na implanty stosowane w kardiologii zabiegowej. Inżynieria Biomateriałów.

[213] Joy-anne N.O., Su Y., Lu X., Kuo P.H., Du J., Zhu D. (2019). Bioactive glass coatings on metallic implants for biomedical applications. Bioact. Mater..

[214] Koleganova V.A., Bernier S.M., Dixon S.J., Rizkalla A.S. (2006). Bioactive glass/polymer composite materials with mechanical properties matching those of cortical bone. J. Biomed. Mater. Res. Part A Off. J. Soc. Biomater. Jpn. Soc. Biomater. Aust. Soc. Biomater. Korean Soc. Biomater..

[215] Hadem H., Mitra A., Ojha A.K., Rajasekaran R., Satpathy B., Das D., Mukherjee S., Dhara S., Das S., Das K. (2024). Electrophoretic Deposition of 58S Bioactive Glass-Polymer Composite Coatings on 316L Stainless Steel: An Optimization for Corrosion, Bioactivity, and Cytocompatibility. ACS Appl. Bio Mater..

[216] Suhaimin I.S., Abu Kassim S., Ahmad Zubir S., Abdullah T.K. (2025). Effect of bioactive glass on the properties of glass/polymer composite scaffold fabricated by solvent casting/particulate leaching technique. J. Thermoplast. Compos. Mater..

[217] Kiefer K., Amlung M., Aktas O.C., de Oliveira P.W., Abdul-Khaliq H. (2016). Novel glass-like coatings for cardiovascular implant application: Preparation, characterization and cellular interaction. Mater. Sci. Eng. C.

[218] Azizli M.J., Lashgari S., Rezaeeparto K., Parham S., Ghadami A., Tayebi L., Vafa E., Asadizadegan M. (2025). Polymer Synergy: Enhancing PA6/PLA Properties with POE-g-MA and Bioactive Glass for Advanced Biomedical Solutions. J. Polym. Environ..

[219] Kucko S.K., Raeman S.M., Keenan T.J. (2023). Current advances in hydroxyapatite-and β-tricalcium phosphate-based composites for biomedical applications: A review. Biomed. Mater. Devices.

[220] Wang Y., Liu C., Song T., Cao Z., Wang T. (2024). 3D printed polycaprolactone/β-tricalcium phosphate/carbon nanotube composite–Physical properties and biocompatibility. Heliyon.

[221] Ferri J.M., Gisbert I., García-Sanoguera D., Reig M.J., Balart R. (2016). The effect of beta-tricalcium phosphate on mechanical and thermal performances of poly (lactic acid). J. Compos. Mater..

[222] Montufar E.B., Casas-Luna M., Horynová M., Tkachenko S., Fohlerová Z., Diaz-De-La-Torre S., Dvořák K., Čelko L., Kaiser J. (2018). High strength, biodegradable and cytocompatible alpha tricalcium phosphate-iron composites for temporal reduction of bone fractures. Acta Biomater..

[223] Nayak V.V. (2023). A Polymer-Bioceramic Composite for Bone Tissue Regeneration. Doctoral Dissertation.

[224] Dobrzyńska-Mizera M., Dodda J.M., Liu X., Knitter M., Oosterbeek R.N., Salinas P., Pozo E., Ferreira A.M., Sadiku E.R. (2024). Engineering of Bioresorbable Polymers for Tissue Engineering and Drug Delivery Applications. Adv. Healthc. Mater..

[225] Tevlek A., Agacik D.T., Aydin H.M. (2020). Stretchable poly (glycerol-sebacate)/β-tricalcium phosphate composites with shape recovery feature by extrusion. J. Appl. Polym. Sci..

[226] Liu W., Ma J., Wang D., Wang P., Zhao J., Wu W., Song W. (2021). Performance modulation and 3D printing parameters optimization of implantable medical tricalcium-silicate/polyetherimide composite. Ceram. Int..

[227] Hashemi M., Ghasemi I., Omrani A., Rostami A., Durán-Valle C.J., Qandalee M. (2025). Biodegradable Shape Memory Nanocomposites Based on PCL/PPC/Graphene: As a Proposal Material for Cardiovascular Stent. J. Polym. Environ..

[228] Wasyluk Ł., Hreniak D., Boiko V., Sobieszczańska B., Bologna E., Zingales M., Pasławski R., Arkowski J., Sareło P., Wawrzyńska M. (2024). Functional Mechanical Behavior and Biocompatible Characteristics of Graphene-Coated Cardiovascular Stents. Int. J. Mol. Sci..

[229] Cheng J., Zheng Y.F. (2013). In vitro study on newly designed biodegradable Fe-X composites (X= W, CNT) prepared by spark plasma sintering. J. Biomed. Mater. Res. Part B Appl. Biomater..

[230] Jones K., Jensen B.D., Bowden A. (2013). Fabrication and testing of planar stent mesh designs using carbon-infiltrated carbon nanotubes. J. Nanotechnol. Eng. Med..

[231] Gerasimenko A.Y., Kurilova U.E., Savelyev M.S., Murashko D.T., Glukhova O.E. (2021). Laser fabrication of composite layers from biopolymers with branched 3D networks of single-walled carbon nanotubes for cardiovascular implants. Compos. Struct..

[232] Paul A., Shao W., Shum-Tim D., Prakash S. (2012). The attenuation of restenosis following arterial gene transfer using carbon nanotube coated stent incorporating TAT/DNAAng1+ Vegf nanoparticles. Biomaterials.

[233] Karagkiozaki V., Karagiannidis P.G., Kalfagiannis N., Kavatzikidou P., Patsalas P., Georgiou D., Logothetidis S. (2012). Novel nanostructured biomaterials: Implications for coronary stent thrombosis. Int. J. Nanomed..

[234] Zhao J.L., Sun B.G., Wen Q.Z., Zhang J.J., Jin W., Xue J.X., Zhuang W.Y. (2007). Effect of early and non-early controlled-release of arsenic-trioxide eluting stents on restenosis inhibition in a canine model. Zhonghua Xin Xue Guan Bing Za Zhi.

[235] Morad Hasely Z., Farahani M.M., Baniassadi M., Chini F., Kajbafzadeh A.M., Kiani A., Baghani M. (2023). Design and fabrication of silicone-silica nanocomposites airway stent. Front. Mater..

[236] Zhao J.L., Sun B.G., Wen Q.Z. (2010). Effects of control-releasing arsenic trioxide-eluting stent on intimal smooth muscle cells and type III collagen in canine coronary artery post-stent model. Zhongguo Zhong Xi Yi Jie He Za Zhi Zhongguo Zhongxiyi Jiehe Zazhi Chin. J. Integr. Tradit. West. Med..

[237] Zhao J.L., Sun B.G., Wen Q.Z. (2008). Effect of Zedoary Turmeric Oil-eluting stents for post-stenting restenosis prevention and treatment. Zhongguo Zhong Xi Yi Jie He Za Zhi Zhongguo Zhongxiyi Jiehe Zazhi Chin. J. Integr. Tradit. West. Med..

[238] Lin J.J., Lin W.C., Li S.D., Lin C.Y., Hsu S.H. (2013). Evaluation of the antibacterial activity and biocompatibility for silver nanoparticles immobilized on nano silicate platelets. ACS Appl. Mater. Interfaces.

[239] Wong W.K., Lai C.H.N., Cheng W.Y., Tung L.H., Chang R.C.C., Leung F.K.C. (2022). Polymer–metal composite healthcare materials: From nano to device scale. J. Compos. Sci..

[240] Toong D.W.Y., Ng J.C.K., Cui F., Leo H.L., Zhong L., Lian S.S., Venkatraman S., Tan L.P., Huang Y.Y., Ang H.Y. (2022). Nanoparticles-reinforced poly-l-lactic acid composite materials as bioresorbable scaffold candidates for coronary stents: Insights from mechanical and finite element analysis. J. Mech. Behav. Biomed. Mater..

[241] Yin R.X., Yang D.Z., Wu J.Z. (2014). Nanoparticle drug-and gene-eluting stents for the prevention and treatment of coronary restenosis. Theranostics.

[242] Kim C.Y., Xu L., Lee E.H., Choa Y.H. (2013). Magnetic silicone composites with uniform nanoparticle dispersion as a biomedical stent coating for hyperthermia. Adv. Polym. Technol..

[243] Ha D.H., Chae S., Lee J.Y., Kim J.Y., Yoon J., Sen T., Lee S.W., Kim H.J., Cho J.H., Cho D.W. (2021). Therapeutic effect of decellularized extracellular matrix-based hydrogel for radiation esophagitis by 3D printed esophageal stent. Biomaterials.

[244] Brown M., Li J., Moraes C., Tabrizian M., Li-Jessen N.Y. (2022). Decellularized extracellular matrix: New promising and challenging biomaterials for regenerative medicine. Biomaterials.

[245] Kang M.K., Heo S.H., Yoon J.K. (2024). In-Stent Re-Endothelialization Strategies: Cells, Extracellular Matrix, and Extracellular Vesicles. Tissue Eng. Part B Rev..

[246] Aubin H., Mas-Moruno C., Iijima M., Schütterle N., Steinbrink M., Assmann A., Gil F.J., Lichtenberg A., Pegueroles M., Akhyari P. (2016). Customized interface biofunctionalization of decellularized extracellular matrix: Toward enhanced endothelialization. Tissue Eng. Part C Methods.

[247] Desai M.S., Lee S.W. (2015). Protein-based functional nanomaterial design for bioengineering applications. Wiley Interdiscip. Rev. Nanomed. Nanobiotechnol..

[248] Su R.S.C., Kim Y., Liu J.C. (2014). Resilin: Protein-based elastomeric biomaterials. Acta Biomater..

[249] Acosta S., Quintanilla-Sierra L., Mbundi L., Reboto V., Rodríguez-Cabello J.C. (2020). Elastin-like recombinamers: Deconstructing and recapitulating the functionality of Extracellular Matrix proteins using recombinant protein polymers. Adv. Funct. Mater..

[250] Patkara S.S., Garcia C.G., Kiicka K.L. (2022). Intrinsically Disordered and Resilin-based Protein Polymers for High-performance Biomaterials. Biomim. Protein Based Elastomers Emerg. Mater. Future.

[251] Srivastava R. (2025). Sustainable Green Biomaterials in Drug Delivery. Sustainable Green Biomaterials as Drug Delivery Systems.

[252] Ichihashi S., Fernández-Colino A., Wolf F., Rojas-González D.M., Kichikawa K., Jockenhoevel S., Schmitz-Rode T., Mela P. (2019). Bio-based covered stents: The potential of biologically derived membranes. Tissue Eng. Part B Rev..

[253] Lau S., Gossen M., Lendlein A. (2021). Designing cardiovascular implants taking in view the endothelial basement membrane. Int. J. Mol. Sci..

[254] Akentjew T.L. (2017). Development of Reinforced and Cell-Laden Vascular Grafts Structurally Inspired by Human Coronary Artery. Ph.D. Thesis.

[255] Heo S., Noh M., Kim Y., Park S. (2024). Stem Cell-Laden Engineered Patch: Advances and Applications in Tissue Regeneration. ACS Appl. Bio Mater..

[256] Park C., Park K.S., Jeong M.J., Kim H.B., Bae I., Lim K.S., Li M.X., Hong Y.J., Han D.K., Joung Y.K. (2021). A robustly supported extracellular matrix improves the intravascular delivery efficacy of endothelial progenitor cells. Adv. Funct. Mater..

[257] Swetha S., Lavanya K., Sruthi R., Selvamurugan N. (2020). An insight into cell-laden 3D-printed constructs for bone tissue engineering. J. Mater. Chem. B.

[258] Limón I., Bedmar J., Fernández-Hernán J.P., Multigner M., Torres B., Rams J., Cifuentes S.C. (2024). A review of additive manufacturing of biodegradable Fe and Zn alloys for medical implants using laser powder bed fusion (LPBF). Materials.

[259] Mayers J., Hofman B., Sobiech I., Kwesiga M.P. (2024). Insights into the biocompatibility of biodegradable metallic molybdenum for cardiovascular applications-a critical review. Front. Bioeng. Biotechnol..

